# Asymmetry in the function and dynamics of the cytosolic group II chaperonin CCT/TRiC

**DOI:** 10.1371/journal.pone.0176054

**Published:** 2017-05-02

**Authors:** Yohei Y. Yamamoto, Yuko Uno, Eiryo Sha, Kentaro Ikegami, Noriyuki Ishii, Naoshi Dohmae, Hiroshi Sekiguchi, Yuji C. Sasaki, Masafumi Yohda

**Affiliations:** 1 Department of Biotechnology and Life Science, Tokyo University of Agriculture and Technology, Koganei, Tokyo, Japan; 2 Biomedical Research Institute, National Institute of Advanced Industrial Science and Technology, Tsukuba, Ibaraki, Japan; 3 The United Graduate School of Agricultural Science, Gifu University, Tsukuba, Ibaraki, Japan; 4 Biomolecular Characterization Unit, RIKEN Center for Sustainable Resource Science, Wako, Saitama, Japan; 5 Japan Synchrotron Radiation Research Institute, Sayo, Hyogo, Japan; 6 Graduate School of Frontier Sciences, University of Tokyo, Kashiwa, Chiba, Japan; 7 Institute of Global Innovation Research, Tokyo University of Agriculture and Technology, Koganei, Tokyo, Japan; Simon Fraser University, CANADA

## Abstract

The eukaryotic group II chaperonin, the chaperonin-containing t-complex polypeptide 1 (CCT), plays an important role in cytosolic proteostasis. It has been estimated that as much as 10% of cytosolic proteins interact with CCT during their folding process. CCT is composed of 8 different paralogous subunits. Due to its complicated structure, molecular and biochemical investigations of CCT have been difficult. In this study, we constructed an expression system for CCT from a thermophilic fungus, *Chaetomium thermophilum* (CtCCT), by using *E*. *coli* as a host. As expected, we obtained recombinant CtCCT with a relatively high yield, and it exhibited fairly high thermal stability. We showed the advantages of the overproduction system by characterizing CtCCT variants containing ATPase-deficient subunits. For diffracted X-ray tracking experiment, we removed all surface exposed cysteine residues, and added cysteine residues at the tip of helical protrusions of selected two subunits. Gold nanocrystals were attached onto CtCCTs via gold-thiol bonds and applied for the analysis by diffracted X-ray tracking. Irrespective of the locations of cysteines, it was shown that ATP binding induces tilting motion followed by rotational motion in the CtCCT molecule, like the archaeal group II chaperonins. When gold nanocrystals were attached onto two subunits in the high ATPase activity hemisphere, the CtCCT complex exhibited a fairly rapid response to the motion. In contrast, the response of CtCCT, which had gold nanocrystals attached to the low-activity hemisphere, was slow. These results clearly support the possibility that ATP-dependent conformational change starts with the high-affinity hemisphere and progresses to the low-affinity hemisphere.

## Introduction

Chaperones are proteins that bind to unfolded or misfolded polypeptides and induce correct folding or facilitate degradation [1, 2]. Most chaperones are heat shock proteins that are expressed under stress conditions [3]. Chaperonin, also known as Hsp60, is one of the most important and ubiquitous chaperones. Chaperonins exist as large oligomeric complexes that comprise two stacked rings, each of which is composed of 7–8 subunits of approximately 60 kDa. Chaperonin captures an unfolded protein in the central cavity of each ring. Folding of the protein is mediated in an ATP-dependent fashion [4, 5].

The chaperonins are subdivided into two families, group I and group II chaperonins. The group I chaperonins exist in eubacteria and organelles, such as mitochondria and chloroplasts. The group I chaperonin system consists of two components: a tetradecameric Hsp60/GroEL and a heptameric co-chaperonin Hsp10/GroES. The structure and functional mechanisms of group I chaperonins have been studied in detail using the chaperonin from *E*. *coli*, GroEL. The co-chaperonin, GroES, binds to GroEL in an ATP-dependent manner and acts as a “lid” to prevent the substrate from escaping while the size of the folding chamber expanded [1, 6]. In contrast, group II chaperonins are found in archaea and in the eukaryotic cytosol. They also consist of two stacked rings, which are each composed of eight 50 to 60 kDa subunits. Different from group I chaperonins, group II chaperonins do not require Hsp10/GroES. Instead, they contain a built-in lid that closes the folding chamber [7–9]. In the open conformation, it captures an unfolded protein and changes to a closed conformation in an ATP-dependent fashion. The conformational transition induces the folding of the encapsulated protein. Finally, group II chaperonins reopen to release the folded protein from the cavity [10, 11]. Group II chaperonins cooperate with a co-chaperone called prefoldin. Prefoldin has a unique six-tentacle, jellyfish-like structure, captures an unfolded protein with its tentacles and transfers it to the group II chaperonin [12].

The protein-folding mechanism mediated by group II chaperonins has been studied by using simpler archaeal homologues [11, 13–16]. Archaeal group II chaperonins are composed of only one or two subunit types. Moreover, the structural stability of group II chaperonins from thermophilic archaea, which are also known as Thermosomes, provided significant advantages in terms of biochemical and biophysical characterizations. The crystal structure of group II chaperonins from *Thermoplasma acidophilum* provided insight into the functional mechanism of group II chaperonins [7, 9]. Therefore, the mechanism of ATP-dependent conformational change for group II chaperonins from an open to closed conformation has been studied using archaeal chaperonins. A series of structures of group II chaperonin from *Methanococcus maripaludis* (MmCpn) in various states provided detailed insights into the domain motions that occur upon nucleotide binding and hydrolysis [17–19]. The conformational change to the closed chaperonin complex begins with the binding of ATP. Compared to the apostate conformation, the apical domains of ATP-bound subunits were rotated by approximately 45°. Detailed kinetic studies have been performed using chaperonins from *Thermococcus* strain KS-1 (TKS1-Cpn) [20]. A UV light-triggered diffracted X-ray tracking (DXT) study with caged-ATP and stopped-flow fluorometry revealed that the lid was partially closed within 1 s after ATP binding, and the closed ring subsequently twisted counterclockwise within 2–6 s, as viewed from the top to bottom of TKS1-Cpn, and the twisted ring reverted to the original open state with a clockwise motion [21]. In addition, the intra-ring and inter-ring communications were studied using various mutant complexes of TKS1-Cpn [22, 23]. Studies of TKS1-Cpn complexes containing mutant subunits have shown that helical protrusion plays an important role in intra-ring cooperativity. The asymmetric ring complex of TKS1-Cpn, which was composed of wild type and ATPase/binding-deficient rings, was constructed using circular permutated covalent mutants. The asymmetric ring complex exhibited ATP-dependent conformational change and protein folding activity as well, which demonstrated that inter-ring communication was dispensable in the reaction cycle of group II chaperonins. The eukaryotic group II chaperonin, the chaperonin-containing t-complex polypeptide 1 (CCT, also known as TRiC, TCP-1 ring Complex), is different from archaeal homologues. Although archaeal group II chaperonins function as general chaperones by capturing denatured protein through hydrophobic interactions, CCT appears to have substrate specificity. Expression of CCT is not induced by stress conditions, but it seems to be required for folding newly synthesized polypeptides. Despite its substrate specificity, CCT is absolutely required for folding many essential proteins, including cytoskeletal proteins such as tubulin and actin, as well as cell cycle regulators, such as CDC20 and CDH1 [24, 25]. It has been estimated that as much as 10% of cytosolic proteins interact with CCT in the course of their folding process [26].

CCT is composed of 8 different paralogue subunits. They are designated CCT1, CCT2, CCT3, CCT4 CCT5, CCT6, CCT7 and CCT8. They are also named CCTα, CCTβ, CCTγ, CCTδ, CCTε, CCTζ, CCTη and CCTθ. CCT subunits share a high amino acid similarity in the phosphate binding loop and the catalytic site but have a low amino acid sequence similarity in the nucleotide binding site, and they may generate variation in ATP binding and hydrolysis capability [27]. The variation among the CCT subunits should be important for the function of CCT. Structures of CCT alone and those with various substrates or co-factors have been solved using cryo-electron microscopy, small-angle X-ray scattering and X ray crystallography [28–34]. However, the structures at an atomic resolution were not sufficient to discriminate subunits in the ring. Thus, even the argument for the subunit arrangement in the ring has yet to be solved. Recently, a hybrid approach utilizing X-ray crystallography and chemical cross-linking mass spectrometry finally yielded a definitive arrangement [35]. The subunit arrangement should be related to its functions.

The difficulty in studying CCT also arises from its complexity in structure. Preparation of a recombinant CCT complex for *in vitro* studies is challenging. Thus, most *in vitro* studies have been conducted using CCT purified from mammalian tissues, including bovine testis or yeast cells expressing one subunit with a tag. Consequently, the role of each subunit has not been rigorously explored. Machida et al succeeded in establishing an efficient expression and purification method for human recombinant CCT using BHK-21 cells [36]. The method appeared to partially improve the productivity of CCT, but it required additional improvement in productivity for detailed biochemical study.

In this study, we constructed an expression system for CCT with *E*. *coli*. For that purpose, we selected CCT from a thermophilic fungus, *Chaetomium thermophilum* (CtCCT), because it showed relatively high thermal stability. As expected, we obtained recombinant CtCCT with a relatively high yield. We evaluated the advantages of the system by characterizing CtCCT variants that contained ATPase-deficient subunits. We examined the dynamics of CtCCT by DXT and revealed an asymmetry in ATP-induced motion in a ring.

## Materials and methods

### Bacterial strains, fungi, proteins, and reagents

The *Escherichia coli* strains used in this study were DH5α and XL10-gold for plasmid propagation and BL21 star (DE3) pRARE (Invitrogen, Carlsbad, CA) for protein expression. *C*. *thermophilum* was obtained from NBRC (Biological Resource Center, National Institute of Technology and Evaluation, Tokyo, Japan). The concentrations of CtCCTs were determined using the Bio-Rad protein assay (Bio-Rad, Hercules, CA) with bovine serum albumin as a standard, and the concentrations are reported as molar concentrations of hexadecamers. The NucleoSpin RNA Plant (Takara Bio Inc., Shiga, Japan) and cDNA Synthesis Kit (Takara Bio Inc., Shiga, Japan) were used for the RNA and cDNA work. KOD-Plus-Neo DNA polymerase was used for gene amplification, and restriction endonucleases were obtained from Toyobo (Osaka, Japan) and New England Biolabs Japan (Tokyo, Japan). The site-directed mutagenesis of CtCCTs was performed using the QuikChange site-directed mutagenesis kit and QuikChange Lightning Multi Site-Directed Mutagenesis Kit (Agilent Technologies, Santa Clara, CA). Nucleotides and other reagents were purchased from Wako Pure Chemical Industries (Osaka, Japan) or Roche and Sigma-Aldrich Japan.

### Cloning, expression and purification of CtCCT variants

The cDNAs for CtCCT subunits were obtained by RT-PCR from mRNA isolated from *Chaetomium thermophilum*. The fungus was cultured with potato-carrot medium at 40°C. The genes of CtCCT subunits were obtained by PCR amplification using the primer pairs (S1 Table). The amplified genes were digested by the enzymes and inserted into the multi cloning site of pET23b (Merck Millipore, Billerica, MA), and their sequences were verified. Then, the sub-cloned genes were amplified with the plasmid sequences from the ribosomal binding site to the stop codon. The fragments were inserted into the pET23b and pET9a (Merck) in the order of CtCCT1-4-2-5 (pET23b-CCT1-4-2-5), and CtCCT3-6-8-7 (pET9a-CCT3-6-8-7), respectively. The Strep-tag sequence was added into the 3’ terminus of the CtCCT3 gene when the fragment was amplified. Their sequences were verified again after the construction.

For the construction of CtCCT^WT^, pET23b-CCT1-4-2-5, and pET9a-CCT3-6-8-7 were simultaneously transformed into *E*. *coli* BL21 star (DE3) pRARE cells. The transformed *E*. *coli* cells were cultured in 2xYT medium containing ampicillin, kanamycin and chloramphenicol. CtCCT^WT^ was purified from the crude extract of *E*. *coli* cells by affinity chromatography, StrepTrap HP (GE Healthcare, Buckinghamshire, UK) was used for the Strep-tag. The eluted fraction was treated with ATP and then purified with the anion exchange column ResourceQ (GE Healthcare). Finally, the CtCCT^WT^ oligomer was purified by size exclusion chromatography using HiLoad 26/600 Superdex 200 pg (GE Healthcare).

For DXT experiments, pET23b-CCT1-4-2-5_ΔCys and pET9a-CCT3-6-8-7_ΔCys were constructed in which the cysteine residues located on the surface were replaced with serine residues, including CtCCT2 C207S/C343S, CtCCT4 C289S, CtCCT5 C151S/C383S, CtCCT6 C281S/C328S, and CtCCT8 C325S. For the expression of CtCCT^C4C8^, the plasmids pET23b-CCT1-4R264C-2-5_ΔCys, which contained a R264C mutation in the CtCCT4, and pET9a-CCT3-6-8K263C-7_ΔCys, which contained a K263C mutation in the CtCCT8, were used. In a similar manner, the pairs of plasmids pET23b-CCT1E266C-4-2S259C-5_ΔCys, pET9a-CCT3-6-8-7_ΔCys, and pET23b-CCT1-4-2-5_ΔCys, pET9a-CCT3-6E254C-8-7S263C_ΔCys were used for the expression of CtCCT^C1C2^ and CtCCT^C6C7^, respectively. These CtCCT variants were purified in the same manner as CtCCT^WT^ described above.

The eight types of plasmids were prepared for the expression of CtCCT HYD variants. The pET23b-HYD1 plasmid included a mutated CtCCT1 gene with an ATP hydrolysis activity-deficient mutation and other wild-type CtCCT4, 2, 5 subunit genes. The pET23b-HYD1 and pET9a-CCT3-6-8-7 plasmids were simultaneously transformed into *E*. *coli* for CtCCT HYD1 expression. In a similar manner, other HYD plasmids were used for the expression of each HYD variant. The mutations induced for the ATP hydrolysis activity deficiency were CtCCT1 D402A, CtCCT2 D389A, CtCCT3 D392A, CtCCT4 D400A, CtCCT5 D410A, CtCCT6 D394A, CtCCT7 D393A, and CtCCT8 D394A. These CtCCT variants were purified in the same manner as CtCCT^WT^ described above.

### Mass spectrometry

The purified CtCCT (2.4 μg) was digested with trypsin (50 ng, TPCK Treated Trypsin, Worthington Biochemical Corp, Lakewood, NJ) in 20 mM Tris-HCl (pH 8.0, 10 μL) containing 0.05% n-Dodecyl β-D-maltoside. An aliquot (2 μL) of the digest was separated on a nanoflow LC (Easy nLC; Thermo Fisher Scientific, Waltham, MA) using a nano–electrospray ionization spray column (NTCC analytical column, C18, φ75 μm × 100 mm, 3 μm; Nikkyo Technos, Tokyo, Japan) eluted with a linear gradient of 0–40% buffer B (100% acetonitrile and 0.1% formic acid) in buffer A (0.1% formic acid in water) at a flow rate of 300 nl/min over 200 min, which was coupled on-line to a Q-Exactive mass spectrometer (Thermo Fisher Scientific) equipped with a nanospray ion source. MS and MS/MS data were acquired using the data-dependent top 5 method. The resulting MS/MS data were searched against an in-house database, including the sequences of all CtCCT variants, using MASCOT (Matrix Science, Boston, MA).

### Transmission electron microscopy

An aliquot of solutions containing CtCCT variants was applied to specimen grids covered with a thin carbon support film that was made hydrophilic by an ion spattering device (HDT-400; JEOL, Tokyo, Japan) and then negatively stained with 1% uranyl acetate for 50 s. The images were recorded with a slow-scan CCD camera (Gatan retractable MultiScan camera; Gatan, Inc., Pleasanton, CA) under low electron dose conditions at magnifications of 25,000 x, and 50,000 x in a transmission electron microscope (Tecnai F20; FEI Company, Eindhoven, The Netherlands) operated at 120 kV. The images were analyzed on computers using DigitalMicrograph (Gatan, Inc.). The CtCCT5 homo-oligomer was negatively stained with 1% gadolinium acetate and observed with a transmission electron microscope (JEM-1400; JEOL, Tokyo, Japan) operating at 120 kV.

### Size exclusion chromatography–multi-angle light scattering (SEC-MALS)

The purified CtCCT complexes were analyzed by SEC–MALS on a WTC-100S5 column (Wyatt Technology, Santa Barbara, CA) equipped with a multi-angle light-scattering detector (MINI DAWN, Wyatt Technology) and a differential refractive index detector (Shodex RI-101, Showa Denko, Tokyo, Japan) in the HPLC system PU-980i (JASCO). A 100 μl aliquot was injected into the column and eluted with buffer (50 mM Tris-HCl pH 8.0, 5 mM MgCl_2_, 100 mM KCl) at 1.0 ml/min. The molecular weight and protein concentration were determined according to the instruction manual (Wyatt Technology).

### Pull-down assay

For this assay, 100 μM of actin (Sigma-Aldrich Japan) and 50 μM of tubulin were denatured in unfolding buffer (6 M guanidine hydrochloride, 50 mM Tris-HCl pH 8.0, 100 mM KCl, 5 mM MgCl_2_) at room temperature for 1 hour. The unfolded proteins were diluted into binding buffer (50 mM Tris-HCl pH 8.0, 100 mM KCl, 5 mM MgCl_2_) and immediately applied to the StrepTrapHP column (GE Healthcare), which was bound with CtCCT^WT^ in advance. The bound proteins were eluted by binding buffer with 2.5 mM D-desthiobiotin. Proteins in the elution mixture were precipitated by the addition of trichloroacetic acid and then separated on 10% SDS gels. The gels were stained with Coomassie Brilliant Blue R-250. Transfer was conducted in transfer buffer (25 mM Tris, 200 mM glycine, 5% methanol) onto a 0.22 μm polyvinylidene difluoride (PVDF) membrane (Millipore). The primary antibody used for actin was the anti-β actin antibody (mAbcam 8224) from Abcam Co. Ltd. (Tokyo, Japan), and the antibody for tubulin was the monoclonal anti-α-tubulin antibody (T5168) from Sigma-Aldrich Japan (Tokyo, Japan). The secondary antibody was an anti-mouse IgG horseradish-peroxidase-linked whole antibody (NA931V, GE Healthcare). The membranes were visualized using the Western BLoT Chemiluminescence HRP Substrate (TaKaRa) and scanned with a Typhoon 8600 (GE Healthcare).

### Luciferase refolding assay

Denatured luciferase was prepared by dissolving luciferase (Sigma) in unfolding buffer (6 M guanidine hydrochloride, 25 mM HEPES-KOH pH 7.4, 50 mM CH_3_CO_2_K, and 5 mM DTT) at room temperature for 1 hour. The 205 nM unfolded luciferase was diluted into refolding buffer (25 mM HEPES-KOH, pH 7.4, 100 mM CH_3_CO_2_K, 10 mM Mg(CH_3_CO_2_)_2_, 2 mM DTT, 1 mM ATP, and 2% DMSO) with or without 400 nM CtCCT variants. For the temperature-dependent assay, at 0, 10, 30, 60, and 90 min, an aliquot of the refolding reaction sample was diluted 1:25 in Luciferase Assay Reagent (Promega, Madison, WI) and luminescence was measured with the GloMax-20/20 Luminometer (Promega). The refolding reaction was performed at 35°C to compare the activity between the HYD variants. At 30 min, an aliquot of the refolding reaction sample was collected, and the luminescence was measured in the same manner as described above.

### Protease sensitivity assay

CtCCT variants (50 nM) were incubated with or without ATP (1 mM) for 30 min at 40°C in TKM buffer with continuous mixing. For the assay with AlFx, 30 mM NaF and Al(NO_3_)_3_ were added along with 0.2 and 1 mM ATP. Digestion with thermolysin (1 ng/μl) was performed for 10 min at 40°C. Proteins in the reaction mixture were precipitated by adding trichloroacetic acid and were then analyzed on 10% SDS gels. The gels were stained with Coomassie Brilliant Blue R-250.

### Diffracted X-ray tracking (DXT)

A 50 μm thick polyimide film (Kapton, Du Pont-Toray, Tokyo, Japan) coated with chromium (10 nm) and gold (25 nm) by vapor deposition was used as a substrate surface for DXT. An aliquot of mutant CtCCT solution (0.2 mg/mL) in MOPS buffer (50 mM MOPS, 100 mM KCl, 5 mM MgCl_2_, pH 7.5) was applied to the gold substrate for 2 h at 4°C. The CtCCT-modified surface was rinsed with the same buffer and reacted with gold nanocrystal solution for 1–2 h at 4°C. The gold nanocrystal-modified CtCCT surface was rinsed with MOPS buffer and stored in the MOPS buffer until further use. An experimental chamber was constructed of sample substrate film with a spacer of polyimide film that was 50 μm thick. The chamber was filled with MOPS buffer containing no ATP or 1 mM ATP or 5 mM caged-ATP for DXT measurement.

The dynamics of CtCCTs were monitored through the trajectories of the Laue spots from the gold nanocrystals, which labeled the chaperonins. White X-rays, 14.0–16.5 keV (Undulator ID gap = 31.0 mm) from the beam line BL40XU (SPring-8, Japan) were used to record the Laue diffraction spots from the gold nanocrystals on CPNs. The size of incident X-ray beams on the sample position was 50 μm in diameter. The gold nanocrystals were exposed to the X-ray beams 90 times and 200 times every 40 ms for 3600 ms and 8000 ms for normal DXT and flash-triggered DXT, respectively. The time-resolved diffraction images were monitored using an X-ray image intensifier (V5445P, Hamamatsu photonics, Hamamatsu, Japan) and a CMOS camera (C11440-10C, Hamamatsu photonics). The specimen-to-sample distance was approximately 100 mm and calibrated by diffraction from the gold film. The sample temperature during DXT was controlled by hot air blowers and maintained at approximately 40°C (TRIAC PID, Leister, Switzerland). A xenon lamp (40 J/flash) was introduced into the X-ray irradiated area for flash-triggered DXT. The sample chamber was replaced after each exposure shot in flash-triggered DXT. Gold nanocrystals were obtained by epitaxial growth on NaCl (100) substrate and were dissolved with detergent (n-Decyl-β-D-maltoside (Dojindo laboratories, Tokyo, Japan), 50 mM MOPS, pH 7.5). The average diameter of the gold nanocrystals was estimated to be 40 nm and was confirmed by AFM images. Custom software written for IGOR Pro (Wavemetrics, Lake Oswego, OR) and a Trackpy package that runs on Python were used to analyze the diffracted spot tracks and trajectories.

### ATPase activity measurement using malachite green

ATPase activities were measured at 30, 35 and 40°C in TKM buffer (50 mM Tris-HCl, pH 7.5, 100 mM KCl, and 25 mM MgCl_2_) containing 1 mM ATP and 20 μg/ml CtCCT variants. Phosphate ion production was measured using the malachite green assay with BIOMOL GREEN^TM^ reagent (Enzo life Sciences).

### ATPase activity measurement with the ATP/NADH coupled assay

ATPase activity was measured at 35°C in HKM buffer (50 mM HEPES-KOH, pH 7.4, 100 mM KCl, and 25 mM MgCl_2_) containing 100 μg/ml pyruvate kinase, 100 μg/ml L-lactate dehydrogenase, 5 mM phosphoenolpyruvate, 0.2 mM nicotinamide adenine dinucleotide (NADH), 2.5 mM DTT, 1 mM ATP and 40 μg/ml CtCCT variants. The rate of steady-state ATP hydrolysis calculated by the measurement of the rate of NADH absorbance decreased at 340 nm.

## Results

### Expression and purification of the CtCCT oligomer

The thermophilic fungus, *Chaetomium thermophilum*, thrives optimally at 50–55°C, and the proteins from the organism were expected to have high stability. Indeed, a number of studies on *C*. *thermophilum* proteins have been reported [37, 38]. Therefore, we decided to try to express CCT from *C*. *thermophilum* (CtCCT). Although a draft genome sequence of *C*. *thermophilum* had been published, the annotation of CCT genes was incomplete. Thus, we annotated CCT genes ourselves (S1 Fig). The amino acid sequences of the CtCCT subunits exhibited significantly high homology scores with the other CCT subunits (S2 Fig). The variation among the CCT subunits should be important for the function of CCT. We confirmed their amino acid sequences to judge whether CtCCT adapted a similar mechanism. Indeed, CtCCT subunits had similar characteristics in the ATP-binding pocket (S3 Fig). This similarity implies that CtCCT functions in the similar manner as other CCTs. After the fungus was grown, the cells were frozen and mRNA was extracted. The cDNAs for CtCCT subunits were obtained through RT-PCR from mRNA.

To express the 8 subunits simultaneously, we constructed two plasmids that each contained 4 subunit genes, including ribosomal binding sites, as an operon (S4 Fig). The genes for CtCCT1, CtCCT4, CtCCT2, and CtCCT5 were cloned into pET23b. The genes for CtCCT3, CtCCT6, CtCCT8, and CtCCT7 were cloned into pET9a in a similar manner. It is known that the CCT subunits CCT4 and CCT5 of *Human* form a homo-oligomer when expressed in *E*. *coli* [39], and recombinant CtCCT5 formed a homo-oligomer in our recombinant system (S5 Fig). To purify the CCT oligomers from the other homo-oligomers, an affinity tag was attached to CtCCT3, which did not form a homo-oligomer.

**Fig 1:**
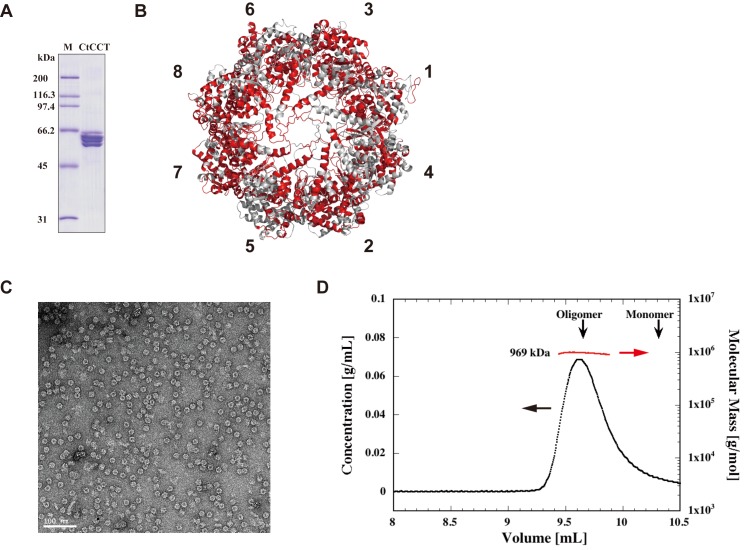
Expression and purification of CtCCT. (A) SDS-PAGE of CtCCT. The purified CtCCT was analyzed by SDS-PAGE followed by staining with Coomassie Brilliant Blue. M, protein marker; CtCCT, purified CtCCT sample. (B) Peptide coverage for CtCCT subunits detected by LC-MS/MS. The matched peptides were shown in red. (C) Transmission electron micrograph of the negatively stained CtCCT. The bar represents 100 nm. (D) Size exclusion chromatography–multi-angle light scattering (SEC-MALS) of CtCCT. The protein concentration (black) was estimated from the differential refractive index, and the molecular mass (red) was determined from the multi-angle light scattering, as described in 'Materials and Methods'.

The plasmids were used for the transformation of *E*. *coli* cells in the presence of ampicillin, kanamycin and chloramphenicol. The CtCCT oligomer was purified from the lysate by affinity chromatography for the Strep-tag attached at the C-terminus of CtCCT3 followed by anion exchange and size exclusion chromatography. SDS-PAGE of the purified CtCCT showed that it is composed of multiple subunits (Fig 1A). The detailed composition was confirmed by liquid chromatography tandem mass spectrometry (LC-MS/MS). The purified CtCCT was digested with a protease, and the peptide fragments were detected and aligned with the peptide sequences of CtCCT subunits. We observed peptide fragments corresponding to all CtCCT subunits. The matching scores for all subunits were large enough to indicate that the purified CtCCT were composed of eight different subunits (Fig 1B, Table 1, S6 Fig). The oligomeric structure was analyzed by transmission electron microscopy and size exclusion chromatography-multi angle light scattering (Fig 1C and 1D). The CtCCT had a double ring-shaped complex that is characteristic of chaperonins. The molecular mass was estimated to be approximately 970 (±49) kDa, and the calculated value from deduced amino sequences was 950 kDa. Consequently, we concluded that the purified CtCCT oligomer (CtCCT^WT^) formed a double ring structure that consisted of 8 paralogous subunits.

**Table 1:** Peptide coverage for CtCCT subunits detected by LC-MS/MS.

Subunit	1	2	3	4	5	6	7	8
Sequence Coverage	63%	53%	69%	46%	46%	58%	61%	80%

### Functional characterization of CtCCT^WT^

**Fig 2:**
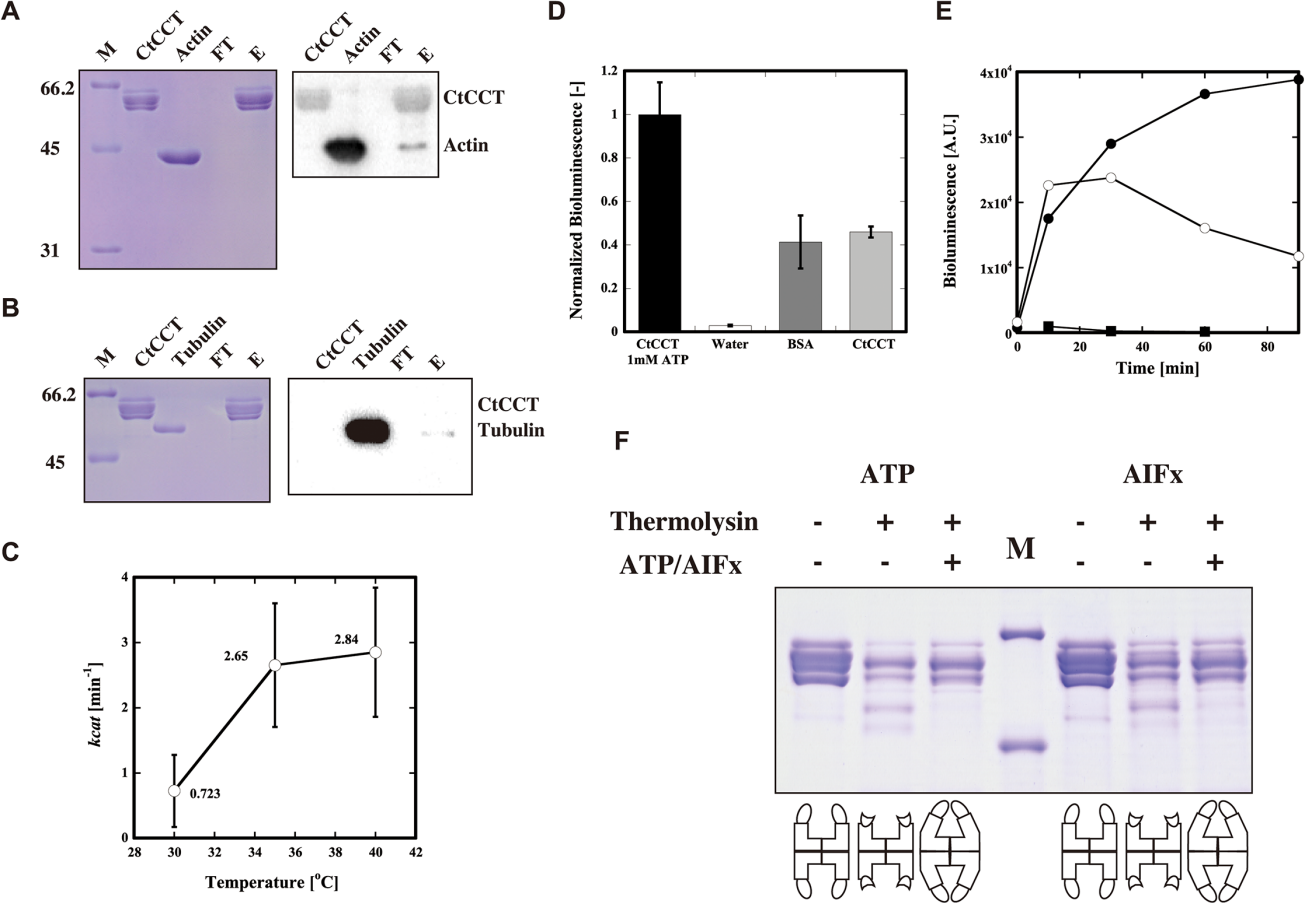
Functional characterization of CtCCT. (A)(B) Interaction of CtCCT with actin and tubulin. Denatured actin and tubulin were applied to a StrepTrap column that was bound with CtCCT in advance. The bound proteins were eluted with CtCCT by elution with D-desthiobiotin. The unbound, washing and elution fractions were separated on SDS-PAGE and visualized by Coomassie brilliant blue staining (Left) or blotting with the antibodies for actin or tubulin (Right). (C) Temperature dependence of ATPase activity. Error bars are the S.E.M of at least three experiments. (D) Luciferase refolding activity of CtCCT. Luciferase refolding was performed at 30°C, and the bioluminescence at 30 min was measured. (E) Temperature dependence of luciferase refolding activity of CtCCT. Luciferase refolding was performed at 30°C (closed circle), 35°C (open circle), and 40°C (closed square), respectively. (F) ATP-dependent conformational change of CtCCT assessed by protease treatment. M, protein marker. The details are described in 'Materials and Methods'.

To confirm whether the recombinant CtCCT^WT^ functioned as CCT, protein binding, ATP hydrolysis, protein folding, and conformational changes were examined. Actin and tubulin are well-defined substrates for CCTs. The binding ability of CtCCT^WT^ with actin and tubulin was assessed by pull-down assay. CtCCT was immobilized on a Strep-Tactin column and acid-denatured actin and tubulin were applied to it. After several washes, CtCCT was eluted by adding 2.5 mM D-desthiobiotin, and the eluted proteins were detected with Coomassie Brilliant Blue stained SDS-PAGE and through Western blots (Fig 2A and 2B). The results clearly showed that CtCCT bound to denatured actin and tubulin. The ATP hydrolysis activity was measured at 30–40°C with a malachite green phosphate assay (Fig 2C). The ATPase activity of CtCCT^WT^ was comparable to that of other CCTs (4.56 /min and 1.146 /min for yeast and bovine CCT, respectively) [40]. It increased with temperature up to 40°C. Protein folding activity was examined by using acid-denatured luciferase as a substrate. CtCCT^WT^ exhibited ATP dependent refolding ability at 30°C (Fig 2D). Unexpectedly, CtCCT^WT^ refolding activity did not increase with the temperature, and no bioluminescence from the refolded luciferase was observed at 40°C (Fig 2E). This outcome could be due to the instability of luciferase at 40°C. We also tried to refold acid-denatured GFP using CtCCT. In contrast to our observation for the archaeal group II chaperonins, CtCCT could not refold the denatured GFP (data not shown), which reflected the narrow substrate specificities of CCT. The ATP-dependent conformational change of the ring was assessed by protease digestion (Fig 2F). Previous studies have shown that group II chaperonin is susceptible to protease digestion in the open conformation [41, 42]. The helical protrusion in the apical domain was the area most susceptible to proteolysis in the entire structure. Similar to archaeal group II chaperonins and also bovine CCT, CtCCTWT was liable to protease digestion in the absence of nucleotide. In the presence of ATP or ATP/Aluminum fluoride(AlFx), the bands for CtCCT^WT^ showed resistance to protease digestion, which indicated that a nucleotide-induced conformational change of CtCCT^WT^ to the closed conformation occurred.

### Detailed analysis of ATP-induced conformational change in CCT using diffracted X-ray tracking

**Fig 3:**
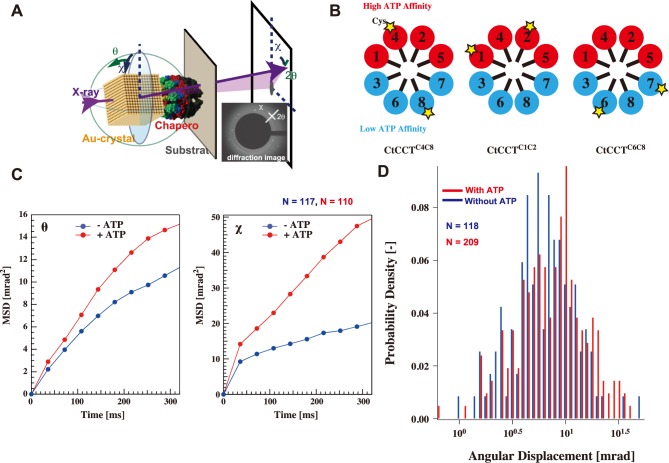
ATP-dependent dynamics of CtCCT analyzed by DXT. (A) Schematic image of the experimental setup for DXT. (B) Schematic drawing of CtCCT variants for the DXT experiments. Subunits are colored according to nucleotide affinity. Red, high; blue, low. The stars represent the subunits into which a Cys residue has been introduced. (C) Mean square angular displacement (MSD) in the θ and χ directions as a function of time with (red) or without (blue) 1 mM ATP. (D) The distribution of absolute angular displacement of CtCCT in the χ direction. With ATP (red), and without ATP (blue). Numbers of analyzed diffraction spots are shown. The details are described in 'Materials and Methods'.

The advantage of our CtCCT expression system is the ease of obtaining CtCCT complexes with the desired mutant subunits. The various CtCCT variants were constructed for further research. Previously, we analyzed the ATP-dependent motion of TKS1-Cpn at the single molecule level by DXT [10, 21, 22]. Gold nanocrystals were used as tracers for the subunit motion of CtCCT. The twisting and tilting of CtCCT corresponded to Laue spots from the gold nanocrystals in the concentric circle (χ) and radial (θ) directions, respectively (Fig 3A). For immobilization on the gold surface and labeling with gold nanocrystals, we constructed mutant CtCCT complexes containing Cys residues at the tip of the helical protrusion. First, the inherent Cys residues located on the molecular surface were changed to Ser residues, including CtCCT2_C207S/C343S, CtCCT4_C289S, CtCCT5_C151S/C383S, CtCCT6_C281S/C328S, and CtCCT8_C325S mutations, to avoid nonspecific interaction with the gold nanocrystals. Based on the Cys-less mutants, we constructed three CtCCT variants, including CtCCT^C4C8^, CtCCT^C1C2^, and CtCCT^C6C7^, for DXT analysis (Fig 3B, S7 Fig). CtCCT^C4C8^ contained Cys residues at the helical protrusions of CtCCT4 (CtCCT4_R264C) and CtCCT8 (CtCCT8_K263C). CtCCT^C1C2^ contained Cys mutations in CtCCT1 (CtCCT1_E266C) and CtCCT2 (CtCCT2_S259C). CtCCT^C6C7^ contained additional Cys residues in CtCCT6 (CtCCT6_E254C) and CtCCT7 (CtCCT7_S263C).

The CtCCT variants were immobilized on a gold-coated substrate surface and labeled with gold nanocrystals through the formation of a gold-thiol bond. CCT1, 2, 4 and 5 were classified as high ATP affinity subunits, and CCT3, 6, 7, and 8 were classified as low ATP affinity subunits. Joachimiak et al speculated that ATP-dependent conformational change started within the high-affinity hemisphere and progressed to the low-affinity hemisphere [43]. CtCCT^C1C2^ and CtCCT^C6C7^ were immobilized on high- and low-affinity hemispheres, respectively. CtCCT^C4C8^ spanned both hemispheres. We expected differences in the dynamics among them. Fig 3C and 3D show the mean square angular displacement (MSD) and histogram of the max angular displacement of χ direction CtCCT^C4C8^ in the absence and in the presence of 1 mM ATP. The MSD curve clearly showed the activation of CtCCT^C4C8^ motion of tilting (θ) and twisting (χ). The histogram of the max angular displacement of the χ direction also showed that the χ directional motion was enhanced. The probabilities of angular displacement greater than 20 mrad in the χ direction were less than 3% in the absence of ATP and approximately 10% in the presence of 1 mM ATP. The p-value for the statistical significance of the difference was estimated to be 0.0267. Therefore, we concluded that CtCCT rotated during the ATP-dependent conformational change in a similar manner as TKS1-Cpn. In the experiment, CtCCTs were incubated in the presence of excess ATP. Thus, there should be a dynamic equilibrium between open and closed states.

**Fig 4:**
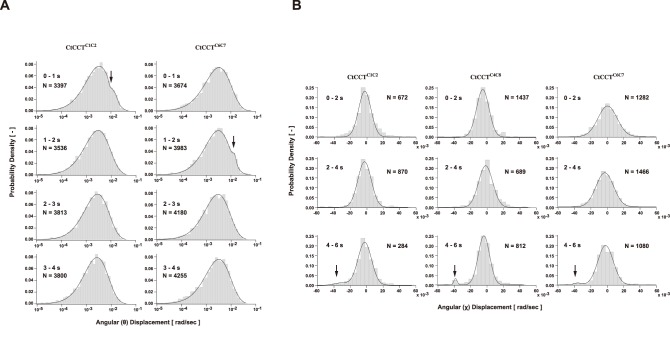
ATP triggered motion of CtCCT analyzed by DXT. ATP triggered motion of CtCCT was analyzed by DXT using caged-ATP. The reaction was started by UV flash. Time-series histograms of the absolute angular velocity in the θ direction (A) and in the χ direction (B) per frame (40 ms). Numbers of analyzed diffraction spots are shown. The details are described in 'Materials and Methods'.

We subsequently attempted to observe the change from an open to a closed state using caged-ATP. Caged-ATP is a derivative of ATP that is inactive and does not bind to the ATP binding site of the chaperonin. UV-light exposure releases the modifying group from the caged-ATP to release active ATP in less than 10 ms [44]. CtCCT was incubated with 5 mM caged-ATP and DXT was started with UV-irradiation to release ATP. To investigate the initial motion induced by ATP binding, the tilting motion, which was observed as θ directional motion, was analyzed. Our previous study suggested that ATP binding to TKS1-Cpn induced the tilting motion that accompanied unsynchronized closure events. Histograms for the displacement each second after UV-light exposure illustrated the absolute angular displacement in the θ direction (Fig 4A). In the analysis of CtCCT^C1C2^, a bimodal distribution was apparent after 1 s of UV light exposure, while normal distributions were observed at the other times. An additional larger peak at approximately 10^−2^ rad in the first histogram corresponded to a larger tilt of the gold nanocrystal in CtCCT and could be explained as unsynchronized closure events because the synchronized closure events corresponded to translational motions that were not reflected as motion in the θ direction in the diffracted images. An additional peak was also observed in CtCCT^C6C7^. Interestingly, the appearance of the additional peak was delayed to between 1 and 2 s after light exposure. The results supported the idea that the conformational change started from the high-affinity hemisphere and progressed to the low-affinity hemisphere. The median values and the pairwise p-values for all of the single molecule distributions calculated by the Wilcoxon rank-sum test are shown in S2 Table. The median values for 0–1 s of CtCCT^C1C2^ and 1–2 s of CtCCT^C6C7^ were the highest probably corresponding to the appearance of additional high mobility peaks.

The ATP hydrolysis triggered further conformational change to the completely closed state. During this conformational change, counterclockwise rotational motion of the ring occurred [30]. Fig 4B shows the histogram constructed by recording the segments of the angular displacement in the χ direction two seconds after UV-light exposure. We observed a significant additional peak at -40 mrad/s in 4–6 s panel in the rotational motion of CtCCT^C4C8^ (Fig 4B). The small additional peak in the 0–2 s panel of CtCCT^C4C8^ was disregarded as the displacement (about 20 mrad/s) was smaller than that in 4–6 s panel (about—40 mrad/s), and also the direction is opposite from that predicted by the structure. From this result, we concluded that counterclockwise rotational motion, corresponding to the closure motion of CCT, started 4–6 s after the UV flush. We could also observe additional small peaks at approximately -40mrad/s in 4–6 s panels of CtCCT^C1C2^ and CtCCT^C6C7^. These results suggest the rotational motion occurred after the conformational motion was completed for all subunits. The pairwise p-values for all of the single molecule distributions calculated by the Wilcoxon rank-sum test are shown in S3 Table.

### Construction and characterization of CtCCT complexes containing ATPase-deficient mutant subunits

**Fig 5:**
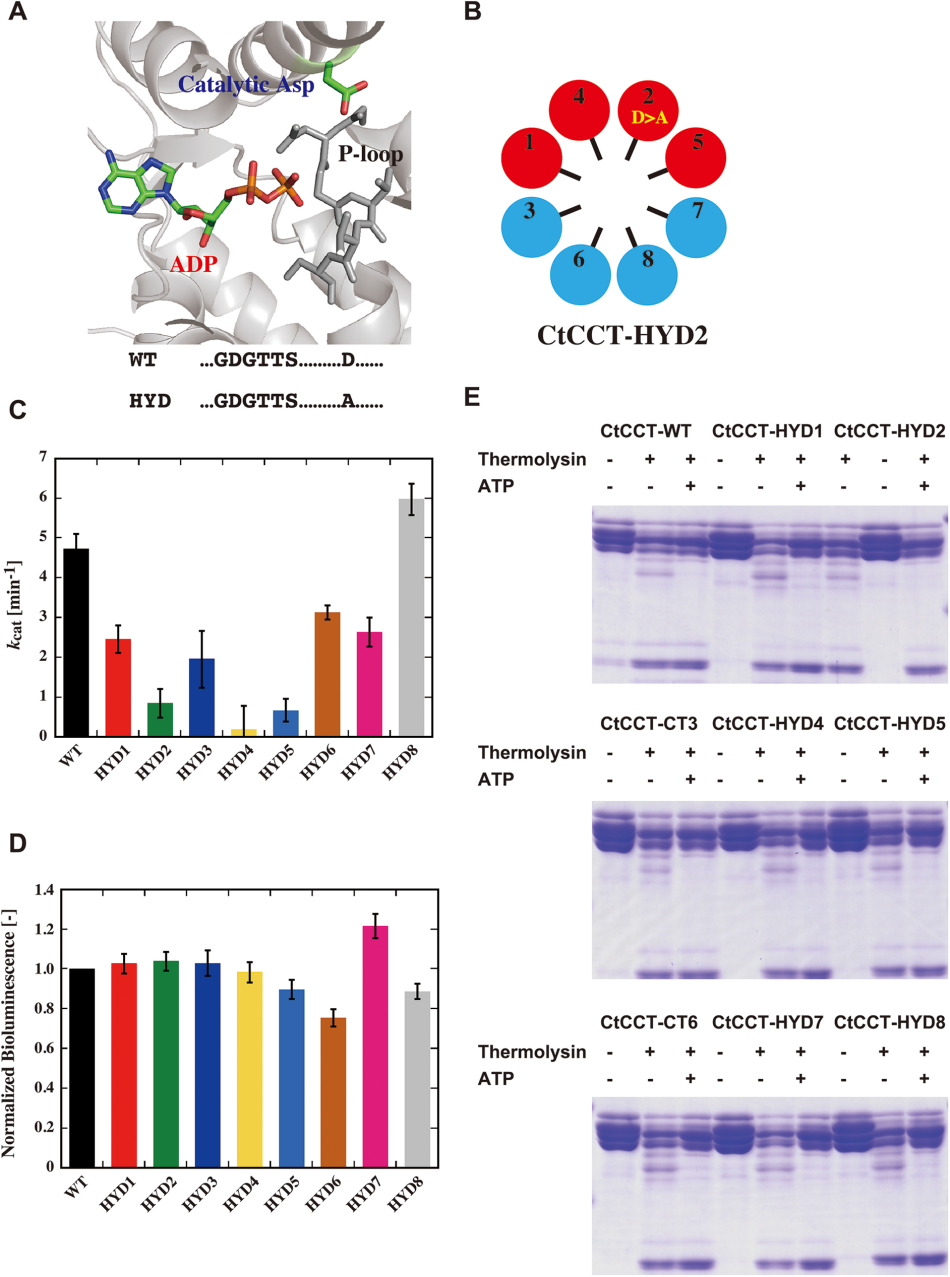
The effect of ATPase deficiency on subunits. (A) CtCCT1 ATP binding site with key residues and mutations involved in ATP hydrolysis highlighted. (B) Schematic drawing of CtCCT HYD variants. Subunits are colored according to nucleotide affinity. Red, high; blue, low. The ATPase deficient mutation was described as D>A. (C) ATP hydrolysis activities of CtCCT variants. Error bars are the S.E.M of at least three experiments. (D) Luciferase refolding activities of CtCCT variants. Error bars are the S.E.M of at least three experiments. (E) Protease digestion assay for the conformational change of CtCCT HYD variants. CtCCT variants incubated with or without ATP (1 mM) were exposed to thermolysin (1 ng/μl) and then analyzed with SDS-PAGE.

To investigate the ATP hydrolysis activity in detail, we constructed CtCCT complexes containing ATPase-deficient subunits. For that purpose, we made mutant subunit genes by replacing the conserved catalytic aspartic acid with alanine. Then, we constructed 8 CtCCT variants that were composed of 7 wild type subunits and one mutant subunit. They were named HYD1, 2, 3, 4, 5, 6, 7, and 8, corresponding to the name of the mutant subunit (Fig 5A and 5B, S8 and S9 Figs). These subunits could assemble into double ring structures similar to the wild type structure.

Then, they were used in an ATP hydrolysis activity assay (Fig 5C). It was expected that an ATPase-deficient mutant in the ring would severely affect the ATPase activity of the CtCCT complex. ATPase deficiency in subunits 2, 4 and 5 significantly impaired the ATPase activity of the CtCCT complexes. The effects of mutations in subunits CtCCT1, 3, 6 and 7 were also not negligible. Curiously, the ATPase activity of HYD8 was greater than that of CtCCT^WT^. Thus, the results were almost consistent with the previous observation that CtCCT1, 2, 4 and 5 constitute the highly active hemisphere [27]. In contrast to the significant effect on ATPase activity, all CtCCT variants maintained almost the same protein folding ability as the wild-type protein (Fig 5D). Among them, the folding ability of HYD6 was the weakest, and the folding ability of HYD7 was the greatest. However, the folding ability did not correlate well with ATPase activities. We then analyzed ATPase-dependent conformational change using protease digestion. Corresponding to folding capabilities, all mutants showed partial protease resistance in the presence of ATP, which was similar to the wild-type protein (Fig 5E).

## Discussion

We succeeded in developing an expression system for CtCCT by using *E*. *coli* as a host. Using this system, we obtained recombinant CtCCT with relatively high yield and developed various mutants. Because CtCCT showed relatively high sequence identity with other CCTs, we used the recombinant CtCCT as a model system for studying the structure and function of CCTs. As expected, CtCCT exhibited almost the same characteristics as other CCTs with relatively high stability. CtCCT interacted with denatured actin and tubulin. CtCCT exhibited ATPase activity and ATP-dependent refolding ability for the denatured luciferase. ATPase activity was maintained up to 40°C. In contrast, the refolding activity of luciferase was not observed at 40°C, which was due to the instability of luciferase at 40°C.

CCT is the most complex chaperone. It is composed of 8 different paralogous subunits that constitute a ring with a fixed arrangement. CCT subunits share high amino acid similarity in the phosphate binding loop and in the catalytic site, but the subunits have low amino acid sequence similarity at the nucleotide binding site, and these differences may generate the variation in ATP binding and hydrolysis [27]. The variation among the CCT subunits appears to be important for the function of CCT. The hetero-oligomeric nature of CCT generated functional and chemical asymmetries different from the other chaperonin systems, which likely provided the basis for substrate specificity.

First, we examined the dynamics of CtCCT at the single molecule level using DXT. In our previous study using TKS1-Cpn [20, 21], the binding of ATP induced a conformational change in each subunit within 1 s. The conformational change appeared as a tilting motion toward the center of the cavity, and it caused TKS1-Cpn to have a partially closed conformation. At this stage, the closure degree of TKS1-Cpn was not enough for protein folding because the substrate was still bound to the TKS1-Cpn subunit and was not released toward the cavity. After the hydrolysis of ATP, TKS1-Cpn achieved a completely closed conformation. The closure event from the partially to completely closed conformation occurred within 2–6 s, and it involved the twisting of the ring in a counter-clockwise direction, as viewed from top to bottom. This rotational motion causes the substrate release from the chaperonin through an intra-ring contact between the adjacent apical domains of helix 11 in one subunit and the release loop for substrate in the adjacent subunit constructing another network interaction [45].

In DXT experiments for CtCCT, we could observe ATP-dependent motion of CtCCT-like TKS1-Cpn. Then we examined the initial motion just after ATP binding using caged-ATP. Similar to the case for TKS1-Cpn, the tilting motion started immediately with the UV flush, and a bimodal distribution appeared afterward (Fig 4A). Importantly, there was a difference the appearance of the bimodal distribution that depended on the location of the Cys residues for binding gold nanocrystals. The CtCCT complex containing Cys residues at the highly active subunits exhibited a relatively rapid response. The response of CtCCT, which had gold nanocrystals attached to the less active hemisphere, was slow. This result clearly supported the idea that the ATP-dependent conformational change started with the high-affinity hemisphere and progressed to the low-affinity hemisphere. Importantly, the rotational motion occurred 4–6 s after the UV flush (Fig 4B). Thus, it was reasonable to think that conformational change with rotation occurred after the completion of inward conformational changes for all subunits. The time is relatively rapid compared to the ATPase cycle. It is reasonable to think that the reaction cycle after the rotation was rate limiting in the reaction cycle of CCT.

The ATP hydrolysis activity of each subunit was estimated from the *in vivo* and the structural studies. Studies for CCT from *S*. *cerevisiae* demonstrated the bipolarity in the ring. Mutations that removed the ability to bind or hydrolyze ATP resulted in severe phenotypes in the four high-affinity CCT subunits (CCT1, CCT2, CCT4 and CCT5) but showed no effect on growth or viability when made in the four low-affinity subunits (CCT3, CCT6, CCT7 and CCT8) [27]. The high- and low-affinity subunits were spatially segregated within two contiguous hemispheres in the ring and generated an asymmetric power stroke that drove the folding cycle. This unusual mode of ATP utilization likely served to orchestrate a directional mechanism underlying the unique ability of CCT to fold substrates of complex eukaryotic proteins. Kalisman et al. estimated the ATPase activities of CCT subunits based on the *in vivo* analyses, the crystal structures and the amino acid sequences [46]. They predicted that CCT1, CCT2, CCT4, CCT5, and CCT7 were active, CCT3 was partially active, and CCT6 and CCT8 were inactive for ATPase activity. Importantly, CCT3 and CCT7 were the inactive subunits in the *in vivo* study [27]. These two studies partially disagreed concerning subunit classification.

We constructed and characterized CtCCT complex variants containing ATPase-deficient mutant subunits. Mutations in 7 of 8 subunits, CtCCT1, 2, 3, 4, 5, 6 and 7, impaired ATPase activity. Among these subunits, the effects of mutations in subunits 2, 4 and 5 were significant. The mutation in CtCCT6 had a relatively marginal effect. In contrast, CtCCT complexes containing ATPase-deficient CCT8 exhibited almost the same or higher ATPase activity compared to the wild-type CCT. The results coincided with the classification made by Kalisman et al. [46].

Then we examined protein refolding ability using denatured luciferase as a substrate. All mutants except the HYD6 mutant exhibited almost the same folding ability as the wild-type CCT. HYD6 had partially impaired protein folding ability. The protease sensitivity assay also demonstrated that all mutant complexes exhibited an ability for ATP-dependent conformational change. These results corresponded with our previous observation on TKS1-Cpn. TKS1-Cpn complexes containing mutant subunits in a ring were capable of protein folding and ATP-dependent conformational change [23].

**Fig 6:**
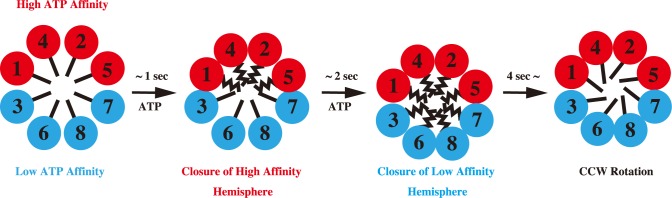
Schematic model of the asymmetric conformational changes in the CCT. Subunits are colored according to nucleotide affinity. Red: high; blue: low. The conformational change may proceed in a sequential fashion owing to the asymmetric usage of ATP by the eukaryotic group II chaperonin. ATP firstly bind to the high ATP affinity hemisphere and induces the conformational change, in less than 1 second. Subsequently, the low ATP affinity hemisphere changes to the closed conformation. Finally, the ring rotates in counter clockwise direction by the cooperative action of the subunits in the ring.

Based on these results, we propose the following conformational change model for CCT (Fig 6). In the absence of ATP, CtCCT is in the open conformation. ATP firstly bind to the high ATP affinity subunits. In less than 1 second, conformational change to the closed conformation occurs in the high ATP affinity hemisphere. Subsequently, the low ATP affinity hemisphere changes to the closed conformation. Finally, the ring rotates in counter clockwise direction by the cooperative action of the subunits in the ring.

Although further studies are needed to reveal the detailed reaction mechanism of CCT, we believe that our system for expressing CtCCT in *E*. *coli* will enhance the study of CCT.

## Supporting information

S1 Fig Nucleotide and amino acid sequences of CtCCT subunits. (PDF)

S2 Fig Sequence Alignments of CCT subunits. (PDF)

S3 Fig ATP binding sites of CtCCT subunits. (A) Alignment of the amino acid in ATP binding site across the eight subunits that comprise the CtCCT. The numbering refer to the ctCCT1 sequence. (B) CtCCT1 active site colored according to conservation scores calculated for CtCCT1 –CtCCT8
(PDF)

S4 Fig Plasmids constructed for expressing CtCCT. (PDF)

S5 Fig Homo-oligomer formation of CtCCT5. (A) EM image of CtCCT homo-oligomer. Black bar represents 100 nm. (B) SEC-MALS analysis of CtCCT5.
(PDF)

S6 Fig Peptide coverage for CtCCT subunits detected by LC-MS/MS. (PDF)

S7 Fig TEM images of CtCCT variants used for DXT experiment. (PDF)

S8 Fig Size exclusion chromatography of CtCCT variants with ATPase deficient mutant subunit. (PDF)

S9 Fig TEM images of CtCCT variants with ATPase deficient mutant subunit. (PDF)

S1 Table Primers used for cloning CtCCT subunits. (PDF)

S2 Table The median values and the pairwise p-values for the single molecule distributions in Fig 4A calculated by the Wilcoxon rank-sum test. (PDF)

S3 Table The pairwise p-values for the single molecule distributions in Fig 4A calculated by the Wilcoxon rank-sum test. (PDF)

## Abbreviations

CCT: cytosolic chaperonin containing TCP-1
TRiC: TCP-1 Ring Complex
CtCCT: CCT of *Chaetomium thermophilum*
CtCCT^WT^: CtCCT wild-type complex
CCTn (n = 1 – 8): subunit n of CCT
CtCCTn (n = 1 – 8): subunit n of CtCCT
HYDn (n = 1 – 8): CtCCT complex containing ATPase-deficient mutation in CtCCTn
TKS1-Cpn: group II chaperonin of hyperthermophilic archaeon *Thermococcus* sp. strain KS-1
MmCpn: group II chaperonin of Methanococcus maripaludis
DXT: diffracted X-ray tracking
MSD: mean square angular displacement
SEC-MALS: Size Exclusion Chromatography – multi-angle light scattering
TEM: transition electron microscopy
GFP: green fluorescent protein
HKM buffer: 50 mM HEPES/KOH pH 7.4, 100 mM KCl and 25 mM MgCl_2_
MOPS buffer: 50 mM MOPS, 100 mM KCl, 5 mM MgCl_2_, pH 7.5
AlFx: Aluminum fluoride
