# Supplementary material for: Asymmetry in the function and dynamics of the cytosolic group II chaperonin CCT/TRiC
Source: PLoS One. 2017 May 2;12(5):e0176054. doi: 10.1371/journal.pone.0176054 (PMC5413064; doi:10.1371/journal.pone.0176054)
Supplement: S1 Fig — (PDF) [file pone.0176054.s001.pdf]

S1 Fig. Nucleotide and amino acid sequences of CtCCT subunits

CtCCT1

ATGGCCGCCATTTTTGAGCAACCAAGGAACGGCACTCTGTTCCTTGGCGGACAGAAGATCTCTGGCGCAGACATTCGGGATCAGAACGTC < 90
M A A I F E Q P R N G T L F L G G Q K I S G A D I R D Q N V

CTTGCTACCCAAGCGATAGCGAATGTCGTTAAGAGCTCTTTTGGCCCCAGCGGCCTCGATAAGATGATGGTCGACGATATCGGTGATGTC < 180
L A T Q A I A N V V K S S F G P S G L D K M M V D D I G D V

ACCGTGACCAACGATGGCGCGACTATCCTGAGCCTCCTCGATGTCGAGCACCCTGCTGGCAAGATCCTGGTCGACCTTGCGCAACAACAA < 270
T V T N D G A T I L S L L D V E H P A G K I L V D L A Q Q Q

GACAAGGAAGTCGGCGACGGAACGACATCAGTTGTTCTGATCGCGGCGGAGCTGCTCAAGCGGGCAAACGACCTGATGAAGAACCGGATA < 360
D K E V G D G T T S V V L I A A E L L K R A N D L M K N R I

CACCCTACCACCATCATTACGGGCTATCGGCTGGCCCTTCGTGAGGCCGTCAAGTACATGAACGAGCACGTCAGCATCAAGGTTGAGAAC < 450
H P T T I I T G Y R L A L R E A V K Y M N E H V S I K V E N

CTGGGGCGCGAGTCGCTGCTCAACATCGCTAAGACTTCCATGTCCAGCAAGATCATCGGCGCTGATGCCGATTTCTTCGCCAACATGGTG < 540
L G R E S L L N I A K T S M S S K I I G A D A D F F A N M V

GTTGACGCCATCCAGGCCGTCAAGACTACCAACAACAAGAACGAGGTCAAGTACCCGGTGAAGGCGGTCAACATCCTCAAGGCTCACGGC < 630
V D A I Q A V K T T N N K N E V K Y P V K A V N I L K A H G

AAGAGTGCCACTGAGTCGATGCTCATCAAGGGCTATGCGCTCAACTGCACTGTTGCCTCGCAGGCCATGAAGACCCGGATAACAGACGCC < 720
K S A T E S M L I K G Y A L N C T V A S Q A M K T R I T D A

AAGATTGCTTGCTTGGATATGAACCTTCAGAAGGAGCGCATGAAGCTGGGTGTTCAGATTACTATTGATGATCCAGAGCAGCTGGAGGCT < 810
K I A C L D M N L Q K E R M K L G V Q I T I D D P E Q L E A

ATTCGGGCTCGGGAAGCAACAATGGTTCTTGAGAGGATCGACATGATTCTCAAGGCTGGCGCCAACGTCATTCTGACCACCAAGGGCATC < 900
I R A R E A T M V L E R I D M I L K A G A N V I L T T K G I

GACGACCTTTGCTTGAAGGCGTTCGTCGAGAAGGGTGCCATGGCTGTTCGCCGCTGCAAGAAGGAGGACCTCCGCCGGATTGCCAAGGCC < 990
D D L C L K A F V E K G A M A V R R C K K E D L R R I A K A

ACTGGCGCCACGCTCCTGGTGACCTTCTCGGATCTTAATGGCGACGAGAAGTTCGAGCCATCTTACCTTGGCCATGCTGAAGAGGTGGTT < 1080
T G A T L L V T F S D L N G D E K F E P S Y L G H A E E V V

CAGGAGCGTATCGCCGACGACGAGTGCATCCTCATCAAGGGTACCAAGGCGCATTCCTCTGCCTCTATCATCCTCCGTGGCCCCAACGAG < 1170
Q E R I A D D E C I L I K G T K A H S S A S I I L R G P N E

TTCACTCTGGACGAGATGGAGCGGTCAGTCCACGACAGCCTGTGTGCTGTCAAGAGGACTTTGGAGAGTGGCAGCATCGTCCCGGGTGGT < 1260
F T L D E M E R S V H D S L C A V K R T L E S G S I V P G G

GGTGCTGTCGAGACGGCCCTGCATATCTTCCTGGAGGAGTTCGCGGGTACCGTTGGCTCGCGCGAGCAGCTCGCAATCGGTGAGTTTGCA < 1350
G A V E T A L H I F L E E F A G T V G S R E Q L A I G E F A

CAGTCGCTGCTTGTCATTCCCAAGACGTTGGCTGTCAATGCGGCCAAGGATGCCTCGGAGCTCGTGGCTCAGCTACGCTCAAGGCACGCC < 1440
Q S L L V I P K T L A V N A A K D A S E L V A Q L R S R H A

CTGTCGCAGCGGATCCAGGAGGGCGAGGGTAGTGAGGACGAGAAGACGATCGCGAAGAAGAAGGCCTACAAGAACTATGGTCTGGATCTG < 1530
L S Q R I Q E G E G S E D E K T I A K K K A Y K N Y G L D L

GTCAGGGGCAAAGTTGTGGATGAGATCAAGGCGGGTGTGGTGGAGCCAAGTATCAGCAAGATTCGCCAGTTGAAGAGCGCGACAGAGGCC < 1620
V R G K V V D E I K A G V V E P S I S K I R Q L K S A T E A

TGCATCGCCATTATGCGGATTGATACCCTGATCAAGCTGGATCCAGAGCCGCAGCCGGAAGATGATGGACACGATCATTAG < 1701
C I A I M R I D T L I K L D P E P Q P E D D G H D H *

CtCCT2

```
ATGTCGTCGTTCAGCCCCACCCAGATCTTCGAGGAGGGAACCACAGAGGAAAAGGGAGAGAATGCTCGTCTTTCCGCCTTCGTCGGCGCC  < 90
M  S  S  F  S  P  T  Q  I  F  E  E  G  T  T  E  E  K  G  E  N  A  R  L  S  A  F  V  G  A

ATCGCCGTCGGCGACCTCGTCAAGAGCACGCTCGGGCCAAAGGGCATGGATAAGATCTTACAGTCTGCGTCAACCGGCGAGATCATGGTG  < 180

ACAAACGATGGCGCGACCATTCTCAAGTCCATCGCTCTCGATAATGCTGCTGCCAAGGTGCTTGTCAACATCTCCAAGGTCCAGGACGAC  < 270
T  N  D  G  A  T  I  L  K  S  I  A  L  D  N  A  A  A  K  V  L  V  N  I  S  K  V  Q  D  D

GAGGTTGGTGATGGTACCACATCGGTCGCCGTGCTTGCTGCCGAGCTGCTGCGCGAGGCCGAGAAGCTTGTGAACCAGAAGATCCATCCC  < 360
E  V  G  D  G  T  T  S  V  A  V  L  A  A  E  L  L  R  E  A  E  K  L  V  N  Q  K  I  H  P

CAGACCATCATCGAGGGTTACCGGATAGCCAGCCAAGCAGCCCTCAAGGCCCTCGAGGCGTCGGCGGTCGACCACAGCAACAACCCTGAG  < 450
Q  T  I  I  E  G  Y  R  I  A  S  Q  A  A  L  K  A  L  E  A  S  A  V  D  H  S  N  N  P  E

CAATTCAAGAAGGACCTGCAGGCCATTGCCCGGACAACACTCAGCTCAAAGGTGCTGGCGCAGGACCGGGACCACTTTGCTAAGCTGGCC  < 540
Q  F  K  K  D  L  Q  A  I  A  R  T  T  L  S  S  K  V  L  A  Q  D  R  D  H  F  A  K  L  A

GTTGAGGCCGTCCTCAGATTGAAGGGCTCGTCCGACCTCAGCCATATCCAGATTATCAAGAAGGCCGGTGGCAAGCTGTGCGAGTCGTAC  < 630
V  E  A  V  L  R  L  K  G  S  S  D  L  S  H  I  Q  I  I  K  K  A  G  G  K  L  C  E  S  Y

CTGGATGAGGGCTTCATTCTCGACAAGAAGATTGGTGTCAACCAGCCGAAGCGCCTGGAGAAGGCCAAAATCCTGATCGCCAACACACCC  < 720
L  D  E  G  F  I  L  D  K  K  I  G  V  N  Q  P  K  R  L  E  K  A  K  I  L  I  A  N  T  P

ATGGACACGGACAAGGTCAAGATCTTTGGCGCCCGGCTCAAGGTCAGCTCGACTAGCAAGCTGGCCGAGCTCGAGCGCGCTGAACGCGAG  < 810
M  D  T  D  K  V  K  I  F  G  A  R  L  K  V  S  S  T  S  K  L  A  E  L  E  R  A  E  R  E

AAGATGAAGGCCAAGGTCGAGAAGATCAAGGCGCACGGCATCAACTGCTTCATCAACCGTCAGCTCATCTACAACTGGCCAGAACAGCTC  < 900
K  M  K  A  K  V  E  K  I  K  A  H  G  I  N  C  F  I  N  R  Q  L  I  Y  N  W  P  E  Q  L

TTCACCGACGCCGGCATCATGTCGATTGAGCACGCTGACTTCGATGGTATCGAGCGGTTGGCCCTCGTCACGGGCGGCGAGATCGCCTCG  < 990
F  T  D  A  G  I  M  S  I  E  H  A  D  F  D  G  I  E  R  L  A  L  V  T  G  G  E  I  A  S

ACCTTCGACCACCCGGAGCAGGTCAAGCTTGGCTACTGCGACCTGATCGAGGAGATTATGATTGGCGAGGACACGCTGATCAAGTTCTCT  < 1080
T  F  D  H  P  E  Q  V  K  L  G  Y  C  D  L  I  E  E  I  M  I  G  E  D  T  L  I  K  F  S

GGTGTCGCTGCCGGCGAGGCCTGCACCATCGTCTTGCGCGGTGCCACCGACCAGCTACTCGACGAAGCCGAGCGCAGTTTGCACGACGCC  < 1170
G  V  A  A  G  E  A  C  T  I  V  L  R  G  A  T  D  Q  L  L  D  E  A  E  R  S  L  H  D  A

CTGGCCGTCTTGTCTCAGATGGTTAAGGAGCCTCGCACGATTCTGGGCGGTGGCTGCGCTGAGATGATCATGGCCAAGGCCGTCGAGGCT  < 1260
L  A  V  L  S  Q  M  V  K  E  P  R  T  I  L  G  G  G  C  A  E  M  I  M  A  K  A  V  E  A

GCTGCGACCCGCGTCGAAGGCAAGAAGCAGACTGCTGTTGGTGCCTTCGCCGTCGCTCTGCGCCAGCTGCCGACAATCCTTGCCGACAAC  < 1350
A  A  T  R  V  E  G  K  K  Q  T  A  V  G  A  F  A  V  A  L  R  Q  L  P  T  I  L  A  D  N

GCTGGTCTTGACTCGGGTGACCTTGTTGCCCGTCTGCGCAAGGCTATCTACGACGGTCTGACCACTTATGGTCTCGACCTGATGACCCCT  < 1440
A  G  L  D  S  G  D  L  V  A  R  L  R  K  A  I  Y  D  G  L  T  T  Y  G  L  D  L  M  T  P

GGCGGCGGCATCGCCGATATGCGCGAGCTGGGTGTCATTGAGAGCTACAAGTTGAAGAGGGCAGTTGTGTCGTCGGCTAGCGAAGCCGCT  < 1530
G  G  G  I  A  D  M  R  E  L  G  V  I  E  S  Y  K  L  K  R  A  V  V  S  S  A  S  E  A  A

GAGCTGCTGTTGCGTGTGGACGATATAATAAGGGCGGCCCCGCGGAAGCGGGAGAAGCACTAA  < 1593
E  L  L  L  R  V  D  D  I  I  R  A  A  P  R  K  R  E  K  H  *
```

CtCCT3

```
ATGCAGGCCCCTGTCTTGGTAATGAACACCCAGAGCGGTGACCGGACGACCGGTCGTCGCGCTCAGATCTCCAACATCGCTGCCGCGAAG  < 90
M  Q  A  P  V  L  V  M  N  T  Q  S  G  D  R  T  T  G  R  R  A  Q  I  S  N  I  A  A  A  K

ACTGTCGCCGACATCATCCGGTCATGCCTCGGCCCCAAGGCCATGCTCAAGATGCTGCTCGACCCCATGGGCGGCATCGTGCTGACCAAC  < 180
T  V  A  D  I  I  R  S  C  L  G  P  K  A  M  L  K  M  L  L  D  P  M  G  G  I  V  L  T  N

GATGGGCATGCTATCCTCCGCGAGATCGAGGTATCGCACCCCGCGGCCAAGAGCATGATTGAGCTGAGCCGGACCCAGGACGAGGAGGTC  < 270
D  G  H  A  I  L  R  E  I  E  V  S  H  P  A  A  K  S  M  I  E  L  S  R  T  Q  D  E  E  V

GGCGATGGGACTACGACGGTTATCATTCTGGCCGGCGAGATCCTCGCCCAGGCCCTGCCCCAGCTCGAGCGCAACATCCACCCCGTCAAC  < 360
G  D  G  T  T  T  V  I  I  L  A  G  E  I  L  A  Q  A  L  P  Q  L  E  R  N  I  H  P  V  N

ATCATCGCCGCCTTCAAGCGCGCCCTCAAGGACGCCCTGGAAATTATCGAGGAGATCTCCATGCCCATCGACGTCAACGACGACAAGGCC  < 450
I  I  A  A  F  K  R  A  L  K  D  A  L  E  I  I  E  E  I  S  M  P  I  D  V  N  D  D  K  A

ATGTACAAGCTGATCAACGCGTCCATCGGCACCAAGTTCGTCTCGCGCTGGTCCGACCTGATGTGCAGCCTAGCGCTGAAGGCCGTGCGC  < 540
M  Y  K  L  I  N  A  S  I  G  T  K  F  V  S  R  W  S  D  L  M  C  S  L  A  L  K  A  V  R

ACCGTGACCTGGGAGGCGAACGGCAAGCGCGAGGTGGACATCAAGCGGTACGCGCGCGTCGAGAAGGTGCCGGGTGGCGAGATCGAGGAC  < 630
T  V  T  W  E  A  N  G  K  R  E  V  D  I  K  R  Y  A  R  V  E  K  V  P  G  G  E  I  E  D

AGCCGCGTGCTCGACGGCGTCATGCTCAACAAGGACATCACGCACCCCAAGATGCGGCGGCGCATCGAGAACCCGCGCATCGTGCTGCTC  < 720
S  R  V  L  D  G  V  M  L  N  K  D  I  T  H  P  K  M  R  R  R  I  E  N  P  R  I  V  L  L

GACTGCCCGCTCGAGTACAAGAAGGGCGAGTCGCAGACCAACATCGAGATCACCAAGGAGGACGACTGGAACCGCATCCTGCAGATCGAG  < 810
D  C  P  L  E  Y  K  K  G  E  S  Q  T  N  I  E  I  T  K  E  D  D  W  N  R  I  L  Q  I  E

GAGGAGCAGGTCAAGGCCATGTGTGACGCCATCCTGGCCGTCAAGCCCGACCTCGTCATCACCGAAAAGGGCGTCTCCGACCTCGCCCAG  < 900
E  E  Q  V  K  A  M  C  D  A  I  L  A  V  K  P  D  L  V  I  T  E  K  G  V  S  D  L  A  Q

CACTACCTGATGAAGGCCAACGTCACCGCCCTGCGCCGCGTCCGCAAGACCGACAACAACCGCATCGCCCGCGCCGTCGGCGCCACCATC  < 990
H  Y  L  M  K  A  N  V  T  A  L  R  R  V  R  K  T  D  N  N  R  I  A  R  A  V  G  A  T  I

GTCAACCGCGTCGAGGACCTGCAGGAGTCCGACGTCGGCACCGGCTGCGGCCTCTTCGAGATCGAGAAGATCGGCGACGAGTACTTCACC  < 1080
V  N  R  V  E  D  L  Q  E  S  D  V  G  T  G  C  G  L  F  E  I  E  K  I  G  D  E  Y  F  T

TTCTTGACCCAATGCAAGAACCCCAAGGCCTGTACCATCCTGCTGCGCGGCCCGTCCAAGGACGTGCTCAACGAGATCGAGCGCAACCTG  < 1170
F  L  T  Q  C  K  N  P  K  A  C  T  I  L  L  R  G  P  S  K  D  V  L  N  E  I  E  R  N  L

CAGGACGCCATGGGCGTCGCGCGCAACGTCATGTTCCATCCGCGCCTGTCGCCCGGCGGCGGCGCGACCGAGATGGCGGTGTCGGTGCGC  < 1260
Q  D  A  M  G  V  A  R  N  V  M  F  H  P  R  L  S  P  G  G  G  A  T  E  M  A  V  S  V  R

CTGGCGCAGATGGCGCGGCAGATTGAGGGCGTCCAGCAGTGGCCTTATAAGGCGGTCGCCGAGGCGCTGGAGGTCATTCCTAGGACGCTG  < 1350
L  A  Q  M  A  R  Q  I  E  G  V  Q  Q  W  P  Y  K  A  V  A  E  A  L  E  V  I  P  R  T  L

GTGCAGAATGCAGGTAAGAGCCCCGTCAGGGTGTTGACGGAGCTCAGGGCCAAGCATGCCGAGGGGAAGCATTCGTTTGGTGTGAATGGC  < 1440
V  Q  N  A  G  K  S  P  V  R  V  L  T  E  L  R  A  K  H  A  E  G  K  H  S  F  G  V  N  G

GATACGGGTGCTGTGGTCGATATGAAGGAGTATGGCGTTTGGGAGCCGCAGGCGATCAAGGTGCAGAGTATGAAGACTGCTATTGAGGCC  < 1530
D  T  G  A  V  V  D  M  K  E  Y  G  V  W  E  P  Q  A  I  K  V  Q  S  M  K  T  A  I  E  A

GCGTGTCTCTTGCTCAGGGTAGACGATATCTGCAGCGCCAAAAAGGCGCAGCCTGGCGTGGGCACTGGTGGTGCTGCGCAGGATGATTAG  < 1620
A  C  L  L  L  R  V  D  D  I  C  S  A  K  K  A  Q  P  G  V  G  T  G  G  A  A  Q  D  D  *
```

CtCCT4

```
ATGGCGACCGCAACACAAGCCGGCAGCGGGGTTACAAACCAAGCATTCAGGGATAAGGAGAAGCCCCTTGCGGTCCGGTCCGCCAACATT  < 90
M  A  T  A  T  Q  A  G  S  G  V  T  N  Q  A  F  R  D  K  E  K  P  L  A  V  R  S  A  N  I

GTCGCCGCCAGAGCTGTCGCCGATGCCATCAGAACATCGCTGGGCCCGCGGGGCATGGACAAGATGATCCGGAGCGGCAAGGGCGAGACC  < 180
V  A  A  R  A  V  A  D  A  I  R  T  S  L  G  P  R  G  M  D  K  M  I  R  S  G  K  G  E  T

ATCATCACGAACGACGGCAGCACGATGCTCAAGAGCATGTCGGTCATGCACCCCACAGCCAAGATGCTCGTTCAGTTGTCTAAGGCCCAG  < 270
I  I  T  N  D  G  S  T  M  L  K  S  M  S  V  M  H  P  T  A  K  M  L  V  Q  L  S  K  A  Q

GACGTCGAGGCCGGTGACGGCACAACCTCGGTCGTGGTCATCTGCGGCAGCCTGTTGGGTGCTGCCGACAGGTTACTACAGAAGGGCATC  < 360
D  V  E  A  G  D  G  T  T  S  V  V  V  I  C  G  S  L  L  G  A  A  D  R  L  L  Q  K  G  I

CACCCATCCGTCATCTCCGAGTCGTTCCAGAGGGCTGCCGCCGCCGCCGTCCAGGTGCTCCACGACATGTCACAACCCATCGCCCTTACA  < 450
H  P  S  V  I  S  E  S  F  Q  R  A  A  A  A  A  V  Q  V  L  H  D  M  S  Q  P  I  A  L  T

GATACCGCCGCCCTTCTCCAGGCCGCCAACACCTCCCTCTCCTCCAAGATCGTTTCGCAGTACTCGAACCTCCTGGGCCCCATGGCCGTC  < 540
D  T  A  A  L  L  Q  A  A  N  T  S  L  S  S  K  I  V  S  Q  Y  S  N  L  L  G  P  M  A  V

AACGCTGTCACCAAGACTATCGACATCAAGACGGCCGACAACGTTGACCTCAAGAACATCCGTGTTATCAAGAAGGTCGGCGGCACGATA  < 630
N  A  V  T  K  T  I  D  I  K  T  A  D  N  V  D  L  K  N  I  R  V  I  K  K  V  G  G  T  I

GAGGACAGCGAGCTGGTCGACGGTCTAGTCTTGACGCAGCCTGTCATCAAGAGCGCGGGAGGGCCTGTACGGATGGAGAAAGCCAGGATT  < 720
E  D  S  E  L  V  D  G  L  V  L  T  Q  P  V  I  K  S  A  G  G  P  V  R  M  E  K  A  R  I

GGCATGATCCAGTTCCAGCTGAGCCCGCCCAAGCCTGATATGGAAAACACCATTCAAGTCAACGACTATCGCCAGATGGACAAGATCGTC  < 810
G  M  I  Q  F  Q  L  S  P  P  K  P  D  M  E  N  T  I  Q  V  N  D  Y  R  Q  M  D  K  I  V

AAAGAGGAGCGCCAGTACCTCCTCAACATGGTCAAGAAAATCAAGAAAGCCAAGTGCAATGTGCTCTTCATCCAGAAGTCCATCCTCCGT  < 900
K  E  E  R  Q  Y  L  L  N  M  V  K  K  I  K  K  A  K  C  N  V  L  F  I  Q  K  S  I  L  R

GACGcCGTCAACGATCTCTCTCTGCACTTcCTGCAGCGCCTGGGCATCCTGGCTGTCAAGGATATTGAGCGTGATGAGGTCGAGTTCATC  < 990
D  A  V  N  D  L  S  L  H  F  L  Q  R  L  G  I  L  A  V  K  D  I  E  R  D  E  V  E  F  I

TGCAAGTCCACTGGCTGCAAGCCCATTGCCGACATCGAGAGCTTCACTGAGGACAAGCTCGGCTCGGCTGACCTCGTCGAGGAGGTGCAC  < 1080
C  K  S  T  G  C  K  P  I  A  D  I  E  S  F  T  E  D  K  L  G  S  A  D  L  V  E  E  V  H

AGCGCCGGCAGCCGCTATGTCAAGGTGACGGGCACAAAGTCGACGGGCAAGACGGTTTCGGTCGTCGTACGTGGTGCCAACAGTTTGATC  < 1170
S  A  G  S  R  Y  V  K  V  T  G  T  K  S  T  G  K  T  V  S  V  V  V  R  G  A  N  S  L  I

TTGGACGAGGCAGAGCGCTCTCTGCACGATGCGCTGTGCGCCGTACGCTGCCTGGTCAAGAAGAAGGCGCTGATCGCTGGTGGTGGCGCG  < 1260
L  D  E  A  E  R  S  L  H  D  A  L  C  A  V  R  C  L  V  K  K  K  A  L  I  A  G  G  G  A

CCCGAGATCGAGATCGCCGCGCAGCTCAACAAGCAGGCCCGGGCACTTTCAGGCACCGAGGCCATCTGCTGGAAGGCCTTTGCAGACGCC  < 1350
P  E  I  E  I  A  A  Q  L  N  K  Q  A  R  A  L  S  G  T  E  A  I  C  W  K  A  F  A  D  A

ATGGAAGTCATCCCGACCACGCTGGCCGAGAACGCTGGTCTTAACCCCATCAAGGTGGTGACAGACCTGCGCCACCGCCACGAGATGGGC  < 1440
M  E  V  I  P  T  T  L  A  E  N  A  G  L  N  P  I  K  V  V  T  D  L  R  H  R  H  E  M  G

GAGAAGAATGCCGGCGTCAGCATCAAGAGCGGCGGTGTCAGCTCCGATATTACCAAGGAGAACGTCCTGCAGCCTTTGCTCGTCAGCACC  < 1530
E  K  N  A  G  V  S  I  K  S  G  G  V  S  S  D  I  T  K  E  N  V  L  Q  P  L  L  V  S  T

AGCGCCATCGAGCTGGCGGCCGAGACGGTCAAGATGATCCTGCGCATTGATGATATCGCCCTGAGCAGGTAA  < 1602
S  A  I  E  L  A  A  E  T  V  K  M  I  L  R  I  D  D  I  A  L  S  R  *
```

CtCCT5

```
ATGGGTTCCATGAATATCGACCTGTCCAACGCGACGGTGATGAAGGATGAGCAGGGCCGGCCCTTCATCGTCGTCAGAGATCAGGGAAAG  < 90
M  G  S  M  N  I  D  L  S  N  A  T  V  M  K  D  E  Q  G  R  P  F  I  V  V  R  D  Q  G  K

AAGAAGCGCCAACATGGCAATGAAGCCGTCAGAGCCCATATCCTCGCCGCCCGCACTGTCGCCAACATTATCAAGACATCACTAGGACCT  < 180
K  K  R  Q  H  G  N  E  A  V  R  A  H  I  L  A  A  R  T  V  A  N  I  I  K  T  S  L  G  P

CGCGGTCTCGACAAGATCCTCATCTCCCCCGACGGCGACATCACAGTAACAAACGATGGAGCTACCATCCTTCAGCAAATGGAGATTACG  < 270
R  G  L  D  K  I  L  I  S  P  D  G  D  I  T  V  T  N  D  G  A  T  I  L  Q  Q  M  E  I  T

AACCACGTTGCTAAGCTGCTGGTTGAGCTGTCCAAGTCACAAGATGACGAGATTGGTGACGGCACCACCGGTGTCGTTGTCCTGGCCGGC  < 360
N  H  V  A  K  L  L  V  E  L  S  K  S  Q  D  D  E  I  G  D  G  T  T  G  V  V  V  L  A  G

GCTCTCCTGGAGCAGGCAGCAGAGCTGATTGACAAGGGCATCCATCCCATCCGTATTGCCGATGGTTACGACCAGGCTTGTGACATTGCC  < 450
A  L  L  E  Q  A  A  E  L  I  D  K  G  I  H  P  I  R  I  A  D  G  Y  D  Q  A  C  D  I  A

TGCGCTGAGCTGGACAGGATCTCAGATGTGATTGAGTTCGACAGGGAGAACACGGAGAACCTCATCAAGGTGGCAAGGACAAGCTTGGGC  < 540
C  A  E  L  D  R  I  S  D  V  I  E  F  D  R  E  N  T  E  N  L  I  K  V  A  R  T  S  L  G

AGCAAGATTGTCTCGAAGGCTCATGACCAATTCGCCAAGATTGCAGTTGATGCCGTCCTGTCAGTTGCCGATCTGGAGCGCAAGGACGTC  < 630
S  K  I  V  S  K  A  H  D  Q  F  A  K  I  A  V  D  A  V  L  S  V  A  D  L  E  R  K  D  V

GACTTCGACCTGATCAAGGTGGACGGTAAGGTCGGCGGTTCGCTCGAAGACACAATGCTCGTCAAGGGCGTTATCATCGACAAGGACTTC  < 720
D  F  D  L  I  K  V  D  G  K  V  G  G  S  L  E  D  T  M  L  V  K  G  V  I  I  D  K  D  F

TCCCATCCCCAGATGCCCTCAGAGGTTCGCGACGCCAAGATCGCCATTTTGACGTGTGCCTTTGAGCCACCCAAGCCAAAGACAAAGCAC  < 810
S  H  P  Q  M  P  S  E  V  R  D  A  K  I  A  I  L  T  C  A  F  E  P  P  K  P  K  T  K  H

AAGCTCGAAATCAGCACCGTGGAAGAGTTCAAAAAGCTACAGAACTATGAGCGGGAGAAGTTCGTCGAGATGATCCAGCAAATAAAGGAC  < 900
K  L  E  I  S  T  V  E  E  F  K  K  L  Q  N  Y  E  R  E  K  F  V  E  M  I  Q  Q  I  K  D

GCCGGCGCGAACCTTGCCATTTGCCAGTGGGGCTTCGATGATGAAGCCAATCACCTGCTCCTTCAGAACAACCTCCCTGCCGTCAGGTGG  < 990
A  G  A  N  L  A  I  C  Q  W  G  F  D  D  E  A  N  H  L  L  L  Q  N  N  L  P  A  V  R  W

GTCGGTGGTCCTGAGATTGAGCTGATTGCCATCGCTACCAACGGCCGGATTGTGCCGCGCTTCGAGGACCTCAGACCTGAGAAGCTTGGT  < 1080
V  G  G  P  E  I  E  L  I  A  I  A  T  N  G  R  I  V  P  R  F  E  D  L  R  P  E  K  L  G

ACCGCCGGCCTCGTGCGGGAAATCACCTTCGGTACGACCCGGGAGAAGATGCTTGTCATTGAGGAGTGTGCCAATACTCGGGCCGTCACC  < 1170
T  A  G  L  V  R  E  I  T  F  G  T  T  R  E  K  M  L  V  I  E  E  C  A  N  T  R  A  V  T

GTTTTCGTCCGGGGTAGCAACAAGATGATCATCGACGAAGCCAAGCGCTCGCTGCACGATGCTCTCTGCGTTGTGCGAAACCTGGTGCGC  < 1260
V  F  V  R  G  S  N  K  M  I  I  D  E  A  K  R  S  L  H  D  A  L  C  V  V  R  N  L  V  R

GACAACCGCGTTGTCTATGGCGGCGGCTCTGCCGAGGTCGCCTGCAGCCTGGCCGTCGAGGACGCGGCTGTCAAGACTCCCGGCCTGGAA  < 1350
D  N  R  V  V  Y  G  G  G  S  A  E  V  A  C  S  L  A  V  E  D  A  A  V  K  T  P  G  L  E

CAGTACGCGATGCGCGCGTTCGCTGAGGCTCTCGACACCATTCCGATGACTCTGGCCGAGAACAGCGGTCTGAACCCCATTGCCACCCTG  < 1440
Q  Y  A  M  R  A  F  A  E  A  L  D  T  I  P  M  T  L  A  E  N  S  G  L  N  P  I  A  T  L

GCCGAAATCAAGAGCCAGCAGGTCAAGGATCCGACCGCCCGTGGCCGTGTCGGTGTCGACTGCATGGGTACTGGCAAGAACAACATGAAG  < 1530
A  E  I  K  S  Q  Q  V  K  D  P  T  A  R  G  R  V  G  V  D  C  M  G  T  G  K  N  N  M  K

GAGGCGTTTGTCATTGATCCACTCATCGGCAAGAAGCAGCAGCTGATGCTGGCAACCCAGCTGTGCCGCATGGTGCTCAAGGTTAACAAT  < 1620
E  A  F  V  I  D  P  L  I  G  K  K  Q  Q  L  M  L  A  T  Q  L  C  R  M  V  L  K  V  N  N

GTCATTGTGTCCGGATCTGGTGAGGAGGAATTTTAA  < 1656
V  I  V  S  G  S  G  E  E  E  F  *
```

CtCCT6

```
ATGTCAGCAGCACAGCTCCTGAACCCGAAAGCGGAGTCGAGGAGGAGGCAAGAGGCGCTCCGTGTCAACATCAGTGCCGGTGAGGGTCTC  < 90
M  S  A  A  Q  L  L  N  P  K  A  E  S  R  R  R  Q  E  A  L  R  V  N  I  S  A  G  E  G  L

CAGGATGTCCTAAAGTCGAATCTGGGCCCCATGGGCACCATCAAGATGCTTGTTGATGGCGCAGGTCAGATCAAACTGACGAAGGACGGC  < 180
Q  D  V  L  K  S  N  L  G  P  M  G  T  I  K  M  L  V  D  G  A  G  Q  I  K  L  T  K  D  G

AATGTCCTGCTCCGCGAGATGCAAATACAAAACCCTACCGCCGTCATGATCGCACGCGCGGCGACGGCCCAGGACGACATCTGCGGCGAT  < 270
N  V  L  L  R  E  M  Q  I  Q  N  P  T  A  V  M  I  A  R  A  A  T  A  Q  D  D  I  C  G  D

GGCACAACATCCGTCGTATTGTTGGTCGGCGAGCTCCTCAAGCAGGCAGACCGCTACATCCAGGAGGGGCTTCACCCTCGTATCATCACC  < 360
G  T  T  S  V  V  L  L  V  G  E  L  L  K  Q  A  D  R  Y  I  Q  E  G  L  H  P  R  I  I  T

GATGGCTTCGAGATTGCTAAGAATGAGGCGCTCAAGTTCCTGGACAAGTTCAAGCTGCCCAAGGATATCGACCGTGAGCTTCTTCTCAAT  < 450
D  G  F  E  I  A  K  N  E  A  L  K  F  L  D  K  F  K  L  P  K  D  I  D  R  E  L  L  L  N

GTTGCCAGGACATCGCTATCGACCAAGCTGAGTTCTAGCCTTGCCCAGCACCTCACACCCAGCATCGTCGATGCCGTTCTCGCCATTTAT  < 540
V  A  R  T  S  L  S  T  K  L  S  S  S  L  A  Q  H  L  T  P  S  I  V  D  A  V  L  A  I  Y

CAGCCCCCGGCTAAGCCTGACCTGCACATGATCGAGATCATGAAGATGCAACACCGGACTGCTTCCGACACACAACTCATTCGCGGCCTT  < 630
Q  P  P  A  K  P  D  L  H  M  I  E  I  M  K  M  Q  H  R  T  A  S  D  T  Q  L  I  R  G  L

GCCCTCGACCACGGCGCTCGGCACCCCGACATGCCCAAGAGGGTAGAAAACGCCTATATTCTGACCCTCAACGTCAGCTTGGAGTACGAG  < 720
A  L  D  H  G  A  R  H  P  D  M  P  K  R  V  E  N  A  Y  I  L  T  L  N  V  S  L  E  Y  E

AAGTCCGAGATCAACTCCGgCTTCTTCTACTCGAGCGCCGAGCAGCGCGACAAGCTGGTGGAGAGCGAACGGAAGTTTGTCGACCTCAAG  < 810
K  S  E  I  N  S  G  F  F  Y  S  S  A  E  Q  R  D  K  L  V  E  S  E  R  K  F  V  D  L  K

CTCAAGAAGATCGTCGAGCTCAAGAAGCAAGTGTGCGGCAACGATCCCAACAAAAACTTCGTCGTCATCAACCAGAAGGGTATCGACCCC  < 900
L  K  K  I  V  E  L  K  K  Q  V  C  G  N  D  P  N  K  N  F  V  V  I  N  Q  K  G  I  D  P

CTGTCTCTCGACGTCCTGGCCAAGAACGGCATCCTGGCTCTCCGGAGAGCCAAGAGGCGCAACATGGAGCGTTTGCAGCTCGTTTGCGGT  < 990
L  S  L  D  V  L  A  K  N  G  I  L  A  L  R  R  A  K  R  R  N  M  E  R  L  Q  L  V  C  G

GGCGTGGCACAGAACAGCGTGGACGACCTGACCCCCGACGTGCTGGGTTGGGCCGGTCTGGTGTACGAGCAGCAGCTGGGCGAGGAGAAG  < 1080
G  V  A  Q  N  S  V  D  D  L  T  P  D  V  L  G  W  A  G  L  V  Y  E  Q  Q  L  G  E  E  K

TACACCTTCGTCGAGGATGTCAAGGACCCCAAGTCGGTGACCATCCTGATCAAGGGTCCCAACCAGCACACCATCACACAGGTGACGGAC  < 1170
Y  T  F  V  E  D  V  K  D  P  K  S  V  T  I  L  I  K  G  P  N  Q  H  T  I  T  Q  V  T  D

GCCGTGCGCGACGGCCTGCGCAGTGTGTACAACTGCATCGTCGACAAGGCTGTTGTGCCCGGCGCCGGCGCCTTCCACGTCGCATGCGCG  < 1260
A  V  R  D  G  L  R  S  V  Y  N  C  I  V  D  K  A  V  V  P  G  A  G  A  F  H  V  A  C  A

GCGCACCTGCGCAGCGATGAATTCCTCAAGGCGGTTAAGGGCAAGGCGAAGTTTGGCGTCGAGGCGTTTGCCGATGCGCTGCTGGTCATT  < 1350
A  H  L  R  S  D  E  F  L  K  A  V  K  G  K  A  K  F  G  V  E  A  F  A  D  A  L  L  V  I

CCCAAGACGCTGGCAGCTAACGCTGGCTTGGACGTGCAGGATGCTGTGGCTCTCCTGCAAGACGAGCTCCGTGCCGGTAATGTGGCCGGC  < 1440
P  K  T  L  A  A  N  A  G  L  D  V  Q  D  A  V  A  L  L  Q  D  E  L  R  A  G  N  V  A  G

ATTGATCTCCAGACGGGCCAGCCTATGGATCCCGTGCTGGAGGGTGTCTTCGACTCGTTCCGCGTCCTGCGCAACTGCATCGCATCCAGC  < 1530
I  D  L  Q  T  G  Q  P  M  D  P  V  L  E  G  V  F  D  S  F  R  V  L  R  N  C  I  A  S  S

TCGAGCATCGCTTCCAACCTGCTGCTGTGCGACGAGCTGCTCAAGGCGCGGCAGATGGGCCGCGGAGGCGGCCCGGGAGGGCCGGGAGGA  < 1620
S  S  I  A  S  N  L  L  L  C  D  E  L  L  K  A  R  Q  M  G  R  G  G  G  P  G  G  P  G  G

ATGGAGGGTGTTGAGGAGTAA  < 1641
M  E  G  V  E  E  *
```

CtCCT7

ATGGCGTTCGCGGGGCAACCCCCAATGATTGTCGTGCTCAAGGAGGGCACCGACACCTCACAGGGCAAGGGCCAGATCCTCTCCAACATC < 90
M A F A G Q P P M I V V L K E G T D T S Q G K G Q I L S N I

AACGCCTGCCTGGCCGTGCAGAGCACGATCAAGTCGACCCTAGGCCCCTATGGCGGCGACCTATTACTCGTCGATCAGAACGGCAAGCAG < 180
N A C L A V Q S T I K S T L G P Y G G D L L L V D Q N G K Q

ACCATCACCAATGACGGAGCTACCGTAATGAAGCTTCTGGACATTGTTCACCCCGCCGCCCGTATCCTCGTCGACATCGCCCGGTCACAA < 270
T I T N D G A T V M K L L D I V H P A A R I L V D I A R S Q

GATGCGGAAGTTGGCGACGGCACGACATCGGTCGTCGTGCTCGCCGGCGAGATTCTGAAGGAGATTAAGGAGCACGTGGAGGCGGGAGTC < 360
D A E V G D G T T S V V V L A G E I L K E I K E H V E A G V

AGCACGCAGATCATAATCAAGGGCCTGAGGAAAGCGGCGTCCATGGCCGTCAACAAGATCAGGGAGGTCGCTATCAACGCAGAAGAGGGA < 450
S T Q I I I K G L R K A A S M A V N K I R E V A I N A E E G

GACCGCATTGATACCCTCCACAAACTAGCGGCGACCGCCATGACCAGCAAGCTTATCAAACGCAACTCTGACTTCTTCACAAAGATGGTT < 540
D R I D T L H K L A A T A M T S K L I K R N S D F F T K M V

GTCGAAGCCGTCCTCTCCCTCGACCAAGACGACCTCAACGAGAAGTTAATAGGCATGAAGAAGATTCCCGGCGGCTCTCTGACCGACTCG < 630
V E A V L S L D Q D D L N E K L I G M K K I P G G S L T D S

ATCTTCGTCAAGGGCGTTGCCTTCAAGAAGACCTTCTCCTACGCTGGCTTCGAGCAGCAGCCCAAGAAGTTCGTCAAGCCCAAGATCTGC < 720
I F V K G V A F K K T F S Y A G F E Q Q P K K F V K P K I C

TGTTTGAACGTCGAGCTCGAGCTCAAGGCCGAGAAGGACAATGCCGAGGTGCGCGTCGAGCAAGTCTCCGAGTACCAGGCCATCGTCGAC < 810
C L N V E L E L K A E K D N A E V R V E Q V S E Y Q A I V D

GCCGAATGGCAAATTATCTACAACAAGCTCGAGGCCATCTACAAGACCGGCGCGAAGGTTGTCCTTTCTAAACTCCCCATTGGCGACCTC < 900
A E W Q I I Y N K L E A I Y K T G A K V V L S K L P I G D L

GCTACGCAATATTTCGCCGACCGCGACATCTTCTGCGCAGGTCGCGTCACCTCCGAGGACATGGAGCGCGTCATCCAGGCAACTGGCGCC < 990
A T Q Y F A D R D I F C A G R V T S E D M E R V I Q A T G A

ACGATCCAGAGCACCTGCTCGGACATCCGCCCCGAGCACCTCGGCACATGCGGGCTCTTCGAAGAGCGCCAGATCGGCGGCGAGCGCTTC < 1080
T I Q S T C S D I R P E H L G T C G L F E E R Q I G G E R F

AACTTCTTCGAGGACTGCCCGGAGGCCAAGACCTGCACTCTCGTCCTGCGCGGCGGTGCCGAGCAGTTCATCGCCGAAGTTGAGCGTTCT < 1170
N F F E D C P E A K T C T L V L R G G A E Q F I A E V E R S

CTGCACGACGCTATCATGATCGTTAAGCGGGCGATCAAGAACAAGACCACCGTCGCCGGCGGCGGCGCCACGGAGATGGAGGTCTCGGCC < 1260
L H D A I M I V K R A I K N K T T V A G G G A T E M E V S A

TACCTGCACCGGTACGCCGACCAGACGGTGCGCAACAAGCAGCAGGCCATCATCAAGAACTTTGCCAAGGCGCTCGAGATTATCCCGCGG < 1350
Y L H R Y A D Q T V R N K Q Q A I I K N F A K A L E I I P R

CAGCTGTGCGACAATGCCGGTTTTGACGCTACCGACATTCTGAACCGGCTGCGCGTGGAGCACAGGCGTGGCAACATCTGGGCGGGTGTG < 1440
Q L C D N A G F D A T D I L N R L R V E H R R G N I W A G V

GACTTCCAGAACGAGGGTGTCGCGGATATGATGGAGAAGTTTGTCTGGGAACCGGCGCTCGTTAAGATCAATGCGATTAATGCGGCTACG < 1530
D F Q N E G V A D M M E K F V W E P A L V K I N A I N A A T

GAGGCGGCGTGCCTTATTCTCAGTGTGGATGAGACTATCCGGAATGAGGAGAGCAAGACCCCGCCGGCGCCGGGCAGCAAGCCCGCGCGT < 1620
E A A C L I L S V D E T I R N E E S K T P P A P G S K P A R

GGTGGCGCGGGACGCGGTCGTGGACGGGGCATGCCGCGGCGGTGA < 1665
G G A G R G R G R G M P R R *

CtCCT8

```
ATGTCCCTTAGCATTCCCGGCGCGCCCAACGCCGGCCTCTTTAAGCAGGGCTACAACAGCTACGATTCCGAAGATGGAGCCGTCCTGCGC  < 90
M  S  L  S  I  P  G  A  P  N  A  G  L  F  K  Q  G  Y  N  S  Y  D  S  E  D  G  A  V  L  R

AACATCGATGCCTGCCGTGCCATCTCGTCGACCGTGCAGACCTCTCTTGGCCCGTACGGCCGCAACAAGGTCGTCATCAACCACCTCGGG  < 180
N  I  D  A  C  R  A  I  S  S  T  V  Q  T  S  L  G  P  Y  G  R  N  K  V  V  I  N  H  L  G

AAGATGATCCTGACCTCCGACGCCGCCACCATCCTGCGCGAGCTCGACGTCGTTCACCCCGCCGCGAAGCTGCTCGTCATGGCCAGCCAA  < 270
K  M  I  L  T  S  D  A  A  T  I  L  R  E  L  D  V  V  H  P  A  A  K  L  L  V  M  A  S  Q

CAGCAGGAGGCCGAGATGGGCGATGCCACGAATCTCGTCATCGTCCTCGCCGGCGAGCTGCTCAGGAAGGCCGAGGACCTGCTGCGCATG  < 360
Q  Q  E  A  E  M  G  D  A  T  N  L  V  I  V  L  A  G  E  L  L  R  K  A  E  D  L  L  R  M

GGCCTCAAGACCTCCGACATTGTCAACGGCTACGAGCGTGCCCAGAAGATCGCACTGGATGCACTGGAAGAGCTCGAGGTCGACAAGGTC  < 450
G  L  K  T  S  D  I  V  N  G  Y  E  R  A  Q  K  I  A  L  D  A  L  E  E  L  E  V  D  K  V

GAGGACCTGCGGAACCCTGAGGAGCTGAAGAAGGCCCTGCGCACCGTCATCGCCAGCAAGCAGAACGGCAGCGAAGACTTCCTTGCCGGC  < 540
E  D  L  R  N  P  E  E  L  K  K  A  L  R  T  V  I  A  S  K  Q  N  G  S  E  D  F  L  A  G

CTCGTCGCTGAGGCCGTCCTCTCCGTGCTGCCCAAGAATCCGGTCAACTTCAACGTCGACAACGTCCGCGTCGTCAAGATCATGGGCGGC  < 630
L  V  A  E  A  V  L  S  V  L  P  K  N  P  V  N  F  N  V  D  N  V  R  V  V  K  I  M  G  G

AGCCTAGACCAGAGCCGCGTCGTCCGCGGTATGGTCTTTAACAAGGAACCGGATGGTGCGGTCAAGAAGGCCCGCAAGGCCAAGGTCGGT  < 720
S  L  D  Q  S  R  V  V  R  G  M  V  F  N  K  E  P  D  G  A  V  K  K  A  R  K  A  K  V  G

GTGTTCACCTGCCCGATCGACATCAGTCAGACGGAGACGAAGGGCACCGTGCTGCTGCACAACGCCAAGGAGATGCTCAACTTCTCCAAG  < 810
V  F  T  C  P  I  D  I  S  Q  T  E  T  K  G  T  V  L  L  H  N  A  K  E  M  L  N  F  S  K

GGCGAGGAGGAGCGGCTCGAGGCCCAGATCAAGGAGCTGCATGACGTCGGTCTCCGCGTTGTCGTCGCCGGCTCCACCGTTGGCGAGCTG  < 900
G  E  E  E  R  L  E  A  Q  I  K  E  L  H  D  V  G  L  R  V  V  V  A  G  S  T  V  G  E  L

GCCATGCACTACCTCAACCGCTACGGCATCCTCGTCATCAAAATCTTCAGCAAGTTCGAGCTGCGCCGTCTGTGCCGCGTCGTCGGCGCT  < 990
A  M  H  Y  L  N  R  Y  G  I  L  V  I  K  I  F  S  K  F  E  L  R  R  L  C  R  V  V  G  A

ACCCCACTCGCTCGCCTCGGCGCCCCCATGCCTGACGAGATGGGTACCATCGACGTTGTTGAGACCCAGGAGATTGGCGGCGACCGTGTG  < 1080
T  P  L  A  R  L  G  A  P  M  P  D  E  M  G  T  I  D  V  V  E  T  Q  E  I  G  G  D  R  V

ACTGTCTTCCGTCAGGAGGACGAGGCCACCCGGACTGCTACCATTGTCCTGCGCGGTGCGACCCAGAACCACCTCGACGATCTCGAGCGT  < 1170
T  V  F  R  Q  E  D  E  A  T  R  T  A  T  I  V  L  R  G  A  T  Q  N  H  L  D  D  L  E  R

GCCGTTGATGATGGTGTCAACGTCATCAAGGCCATCACCAAGGATGCCCGTCTCGTGCCCGGCGCTGGCGCCACCGAGATTGAGCTGGTT  < 1260
A  V  D  D  G  V  N  V  I  K  A  I  T  K  D  A  R  L  V  P  G  A  G  A  T  E  I  E  L  V

GACAGGATACAGGCTGCTGCTGATAAGACCCCCGGTCTGGCGCAGTACTCGATCAAGAAGTACGGCGAGGCCTTTGAGGTCGTGCCGCGT  < 1350
D  R  I  Q  A  A  A  D  K  T  P  G  L  A  Q  Y  S  I  K  K  Y  G  E  A  F  E  V  V  P  R

ACGCTAGCCGAGAGTGCTGGTCTGGATGCTACTGAGGTCGTCAGCAGGCTGTATGCTGCACACCAGAAGAAGGATGGCTGGACTACTGGT  < 1440
T  L  A  E  S  A  G  L  D  A  T  E  V  V  S  R  L  Y  A  A  H  Q  K  K  D  G  W  T  T  G

GTGGATATCGAGAACCAGGACAATACCGGTGTCCTCGACGCCGAGGACGAGGGTATCCTTGACCTGCTCTCCTCCAAGCAATGGGCCATC  < 1530
V  D  I  E  N  Q  D  N  T  G  V  L  D  A  E  D  E  G  I  L  D  L  L  S  S  K  Q  W  A  I

AAGCTGGCCACTGAGGCTGCCCGCACCGTCCTGTCCGTCGACCAGATCATCGTCGCCCGCCAGGCTGGTGGGCCAAAGCCTCCTGGACCC  < 1620
K  L  A  T  E  A  A  R  T  V  L  S  V  D  Q  I  I  V  A  R  Q  A  G  G  P  K  P  P  G  P

AACCCGAACTGGGACGAAGACTGA  < 1644
N  P  N  W  D  E  D  *
```
