# Supplementary material for: Asymmetry in the function and dynamics of the cytosolic group II chaperonin CCT/TRiC
Source: PLoS One. 2017 May 2;12(5):e0176054. doi: 10.1371/journal.pone.0176054 (PMC5413064; doi:10.1371/journal.pone.0176054)
Supplement: S2 Fig — (PDF) [file pone.0176054.s002.pdf]

S2 Fig. Sequence Alignments of CCT subunits

CCT1

```
HUMAN         --------MEGPLSVFGDRSTGETIRSQNVMAAASIANIVKSSLGPVGLDKMLVDDIGDV
CtCCT1        MAAIFEQPRNGTLFLGGQKISGADIRDQNVLATQAIANVVKSSFGPSGLDKMMVDDIGDV
YEAST         MSQLFNNSRSDTLFLGGEKISGDDIRNQNVLATMAVANVVKSSLGPVGLDKMLVDDIGDF
                       .. * : *:: :*  **.***:*: ::**:****:** *****:******.

HUMAN         TITNDGATILKLLEVEHPAAKVLCELADLQDKEVGDGTTSVVIIAAELLKNADELVKQKI
CtCCT1        TVTNDGATILSLLDVEHPAGKILVDLAQQQDKEVGDGTTSVVLIAAELLKRANDLMKNRI
YEAST         TVTNDGATILSLLDVQHPAGKILVELAQQQDREIGDGTTSVVIIASELLKRANELVKNKI
              *:********.**:*:***.*:* :**: **:*:********:**:****.*::*:*::*

HUMAN         HPTSVISGYRLACKEAVRYINENLIVNTDELGRDCLINAAKTSMSSKIIGINGDFFANMV
CtCCT1        HPTTIITGYRLALREAVKYMNEHVSIKVENLGRESLLNIAKTSMSSKIIGADADFFANMV
YEAST         HPTTIITGFRVALREAIRFINEVLSTSVDTLGKETLINIAKTSMSSKIIGADSDFFSNMV
              ***::*:*:*:* :**::::** :  ..: **:: *:* *********** :.***:***

HUMAN         VDAVLAIKYTDIRGQPRYPVNSVNILKAHGRSQMESMLISGYALNCVVGSQGMPKRIVN-
CtCCT1        VDAIQAVKTTNNKNEVKYPVKAVNILKAHGKSATESMLIKGYALNCTVASQAMKTRITD-
YEAST         VDALLAVKTQNSKGEIKYPVKAVNVLKAHGKSATESLLVPGYALNCTVASQAMPKRIAGG
              ***: *:*  : :.: :***::**:*****:*  **:*: ******.*.**.* .**..

HUMAN         -AKIACLDFSLQKTKMKLGVQVVITDPEKLDQIRQRESDITKERIQKILATGANVILTTG
CtCCT1        -AKIACLDMNLQKERMKLGVQITIDDPEQLEAIRAREATMVLERIDMILKAGANVILTTK
YEAST         NVKIACLDLNLQKARMAMGVQINIDDPEQLEQIRKREAGIVLERVKKIIDAGAQVVLTTK
               .******:.*** :* :***: * ***:*: ** **: :. **:. *: :**:*:***

HUMAN         GIDDMCLKYFVEAGAMAVRRVLKRDLKRIAKASGATILSTLANLEGEETFEAAMLGQAEE
CtCCT1        GIDDLCLKAFVEKGAMAVRRCKKEDLRRIAKATGATLLVTFSDLNGDEKFEPSYLGHAEE
YEAST         GIDDLCLKEFVEAKIMGVRRCKKEDLRRIARATGATLVSSMSNLEGEETFESSYLGLCDE
              ****:*** ***   *.***  *.**:***:*:***:: ::::*:*:*.** : ** .:*

HUMAN         VVQERICDDELILIKNTKARTSASIILRGANDFMCDEMERSLHDALCVVKRVLESKSVVP
CtCCT1        VVQERIADDECILIKGTKAHSSASIILRGPNEFTLDEMERSVHDSLCAVKRTLESGSIVP
YEAST         VVQAKFSDDECILIKGTSKHSSSSIILRGANDYSLDEMERSLHDSLSVVKRTLESGNVVP
              *** ::.*** ****.*. ::*:****** *::  ******:**:*..***.*** .:**

HUMAN         GGGAVEAALSIYLENYATSMGSREQLAIAEFARSLLVIPNTLAVNAAQDSTDLVAKLRAF
CtCCT1        GGGAVETALHIFLEEFAGTVGSREQLAIGEFAQSLLVIPKTLAVNAAKDASELVAQLRSR
YEAST         GGGCVEAALNIYLDNFATTVGSREQLAIAEFAAALLIIPKTLAVNAAKDSSELVAKLRSY
              ***.**:** *:*:::* ::********.*** :**:**:*******:*:::***:**:

HUMAN         HNEAQVN------------PERKNLKWIGLDLSNGKPRDNKQAGVFEPTIVKVKSLKFAT
CtCCT1        HALSQRIQEGEGSEDEKTIAKKKAYKNYGLDLVRGKVVDEIKAGVVEPSISKIRQLKSAT
YEAST         HAASQMAKP--------EDVKRRSYRNYGLDLIRGKIVDEIHAGVLEPTISKVKSLKSAL
              *  :*               :::  :  **** .**  *: :***.**:* *::.** *

HUMAN         EAAITILRIDDLIKLHPESKDDKHGSYEDAVHSGALND
CtCCT1        EACIAIMRIDTLIKLDPEPQPEDDGHDH----------
YEAST         EACVAILRIDTMITVDPEPPKED-PHDH----------
              **.::*:*** :*.:.**   :.    .
```

```
Alignment Scores
1: HUMAN        100.00    63.92    62.39
2: CtCCT1        63.92   100.00    72.89
3: YEAST         62.39    72.89   100.00
```

CCT2

```
HUMAN       MASLSLAPVNIFKAGADEERAETARLTSFIGAIAIGDLVKSTLGPKGMDKILLSSGRDAS
CtCCT2      --MSSFSPTQIFEEGTTEEKGENARLSAFVGAIAVGDLVKSTLGPKGMDKILQSA-STGE
YEAST       ------MSVQIFGDQVTEERAENARLSAFVGAIAVGDLVKSTLGPKGMDKLLQSA-SSNT
                    .:**   . **:.*.***::*:****:***************:* *:

HUMAN       LMVTNDGATILKNIGVDNPAAKVLVDMSRVQDDEVGDGTTSVTVLAAELLREAESLIAK-
CtCCT2      IMVTNDGATILKSIALDNAAAKVLVNISKVQDDEVGDGTTSVAVLAAELLREAEKLVNQ-
YEAST       CMVTNDGATILKSIPLDNPAAKVLVNISKVQDDEVGDGTTSVTVLSAELLREAEKLIDQS
             ***********.* :** ******::*:*************:**:********.*: :

HUMAN       KIHPQTIIAGWREATKAAREALLSSAVDHGSDEVKFRQDLMNIAGTTLSSKLLTHHKDHF
CtCCT2      KIHPQTIIEGYRIASQAALKALEASAVDHSNNPEQFKKDLQAIARTTLSSKVLAQDRDHF
YEAST       KIHPQTIIEGYRLASAAALDALTKAAVDNSHDKTMFREDLIHIAKTTLSSKILSQDKDHF
            ******** *:* *: ** .**  :***:. :   *::**  ** ******:*::.:***

HUMAN       TKLAVEAVLRLKGSGNLEAIHIIKKLGGSLADSYLDEGFLLDKKIGVNQPKRIENAKILI
CtCCT2      AKLAVEAVLRLKGSSDLSHIQIIKKAGGKLCESYLDEGFILDKKIGVNQPKRLEKAKILI
YEAST       AELATNAILRLKGSTNLEHIQIIKILGGKLSDSFLDEGFILAKKFGNNQPKRIENAKILI
            ::**.:*:****** :*. *:***  **.*.:*:*****:* **:* *****:*:*****

HUMAN       ANTGMDTDKIKIFGSRVRVDSTAKVAEIEHAEKEKMKEKVERILKHGINCFINRQLIYNY
CtCCT2      ANTPMDTDKVKIFGARLKVSSTSKLAELERAEREKMKAKVEKIKAHGINCFINRQLIYNW
YEAST       ANTTLDTDKVKIFGTKFKVDSTAKLAQLEKAEREKMKNKIAKISKFGINTFINRQLIYDY
            *** :****:****::.:*.**:*:*::*:**:**** *: :*  .*** ********::

HUMAN       PEQLFGAAGVMAIEHADFAGVERLALVTGGEIASTFDHPELVKLGSCKLIEEVMIGEDKL
CtCCT2      PEQLFTDAGIMSIEHADFDGIERLALVTGGEIASTFDHPEQVKLGYCDLIEEIMIGEDTL
YEAST       PEQLFTDLGINSIEHADFEGVERLALVTGGEVVSTFDEPSKCKLGECDVIEEIMLGEQPF
            *****   *: :****** *:**********:.****.*.  *** *.:***:*:**: :

HUMAN       IHFSGVALGEACTIVLRGATQQILDEAERSLHDALCVLAQTVKDSRTVYGGGCSEMLMAH
CtCCT2      IKFSGVAAGEACTIVLRGATDQLLDEAERSLHDALAVLSQMVKEPRTILGGGCAEMIMAK
YEAST       LKFSGCKAGEACTIVLRGATDQTLDEAERSLHDALSVLSQTTKETRTVLGGGCAEMVMSK
            ::***   ************:* ************.**:* .*: **: ****:**:*::

HUMAN       AVTQLANRTPGKEAVAMESYAKALRMLPTIIADNAGYDSADLVAQLRAAHSEGNTTAGLD
CtCCT2      AVEAAATRVEGKKQTAVGAFAVALRQLPTILADNAGLDSGDLVARLRKAIYDGLTTYGLD
YEAST       AVDTEAQNIDGKKSLAVEAFARALRQLPTILADNAGFDSSELVSKLRSSIYNGISTSGLD
            **   * .  **:  *: ::* *** ****:***** **.:**::** :  :* :* ***

HUMAN       MR--EGTIGDMAILGITESFQVKRQVLLSAAEAAEVILRVDNIIKAAPRKRVPDHHPC
CtCCT2      LMTPGGGIADMRELGVIESYKLKRAVVSSASEAAELLLRVDDIIRAAPRKREKH----
YEAST       LN--NGTIADMRQLGIVESYKLKRAVVSSASEAAEVLLRVDNIIRARPRTANRQHM--
            :    * *.**  **: **:::** *: **:****::****:**:* **.    .
```

```
Alignment Scores
1: HUMAN        100.00    69.13    65.59
2: CtCCT2        69.13   100.00    73.66
3: YEAST         65.59    73.66   100.00
```

CCT3

```
HUMAN        MMGHRPVLVLSQNTKRESGRKVQSGNINAAKTIADIIRTCLGPKSMMKMLLDPMGGIVMT
CtCCT3       -MQAPVLVMNTQSGDRTTGRRAQISNIAAAKTVADIIRSCLGPKAMLKMLLDPMGGIVLT
YEAST        -MQAPVVFMNA-SQERTTGRQAQISNITAAKAVADVIRTCLGPKAMLKMLLDPMGGLVLT
              *    :.: : . .* :**:.* .** ***::**:**:*****:*:*********:*:*

HUMAN        NDGNAILREIQVQHPAAKSMIEISRTQDEEVGDGTTSVIILAGEMLSVAEH-FLEQQMHP
CtCCT3       NDGHAILREIEVSHPAAKSMIELSRTQDEEVGDGTTTVIILAGEILAQALPQ-LERNIHP
YEAST        NDGHAILREIDVAHPAAKSMLELSRTQDEEVGDGTTTVIILAGEILAQCAPYLIEKNIHP
             ***:******:* *******:*:*************:*******:*: .    :*:::**

HUMAN        TVVISAYRKALDDMISTLKKISIPVDISDSDMMLNIINSSITTKAISRWSSLACNIALDA
CtCCT3       VNIIAAFKRALKDALEIIEEISMPIDVNDDKAMYKLINASIGTKFVSRWSDLMCSLALKA
YEAST        VIIIQALKKALTDALEVIKQVSKPVDVENDAAMKKLIQASIGTKYVIHWSEKMCELALDA
             . :* * ::** * :. ::::* *:*:.:.  * ::*::** ** : :**.  *.:**.*

HUMAN        VKMVQF------EENGRKEIDIKKYARVEKIPGGIIEDSCVLRGVMINKDVTHPRMRRYI
CtCCT3       VRTVTW------EANGKREVDIKRYARVEKVPGGEIEDSRVLDGVMLNKDITHPKMRRRI
YEAST        VKTVRKDLGQTVEGEPNFEIDIKRYVRVEKIPGGDVLDSRVLKGVLLNKDVVHPKMSRHI
             *: *        * : . *:***:*.****:*** : ** ** **::***:.**:* * *

HUMAN        KNPRIVLLDSSLEYKKGESQTDIEITREEDFTRILQMEEEYIQQLCEDIIQLKPDVVITE
CtCCT3       ENPRIVLLDCPLEYKKGESQTNIEITKEDDWNRILQIEEEQVKAMCDAILAVKPDLVITE
YEAST        ENPRVVLLDCPLEYKKGESQTNIEIEKEEDWNRILQIEEEQVQLMCEQILAVRPTLVITE
             :***:****. **********:*** :*:*:.****:*** :: :*: *: ::* :****

HUMAN        KGISDLAQHYLMRANITAIRRVRKTDNNRIARACGARIVSRPEELREDDVGTGAGLLEIK
CtCCT3       KGVSDLAQHYLMKANVTALRRVRKTDNNRIARAVGATIVNRVEDLQESDVGTGCGLFEIE
YEAST        KGVSDLAQHYLLKGGCSVLRRVKKSDNNRIARVTGATIVNRVEDLKESDVGTNCGLFKVE
             **:********::.. :.:***:*:*******. ** **.* *:*:*.****..**::::

HUMAN        KIGDEYFTFITDCKDPKACTILLRGASKEILSEVERNLQDAMQVCRNVLLDPQLVPGGGA
CtCCT3       KIGDEYFTFLTQCKNPKACTILLRGPSKDVLNEIERNLQDAMGVARNVMFHPRLSPGGGA
YEAST        MIGDEYFSFLDNCKEPKACTIMLRGGSKDILNEIDRNLQDAMAVARNVMLSPSLSPGGGA
              ******:*: :**:******:*** **::*.*::******* *.***:: * * *****

HUMAN        SEMAVAHALTEKSKAMTGVEQWPYRAVAQALEVIPRTLIQNCGASTIRLLTSLRAKHTQE
CtCCT3       TEMAVSVRLAQMARQIEGVQQWPYKAVAEALEVIPRTLVQNAGKSPVRVLTELRAKHAEG
YEAST        TEMAVSVKLAEKAKQLEGIQQWPYQAVADAMECIPRTLIQNAGGDPIRLLSQLRAKHAQG
             :****:  *:: :: : *::****:***:*:* *****:**.* . :*:*:.*****::

HUMAN        NCETWGVNGETGTLVDMKELGIWEPLAVKLQTYKTAVETAVLLLRIDDIVSGHKKKGDDQ
CtCCT3       K-HSFGVNGDTGAVVDMKEYGVWEPQAIKVQSMKTAIEAACLLLRVDDICSAKKAQPGVG
YEAST        N-FTTGIDGDKGKIVDMVSYGIWEPEVIKQQSVKTAIESACLLLRVDDIVSGVRKQE---
             :  : *::*:.* :*** . *:*** .:* *: ***:*:* ****:*** *. : :

HUMAN        SRQGGAPDAGQE
CtCCT3       T--GGAAQDD--
YEAST        ______
```

Alignment Scores

1: HUMAN	100.00	63.94	59.96
2: CtCCT3	63.94	100.00	73.62
3: YEAST	59.96	73.62	100.00

CCT4

```
HUMAN          MPENVAPRSGATAGAAGGRGKGAYQDRDKPAQIRFSNISAAKAVADAIRTSLGPKGMDKM
CtCCT4         --------MATATQAGSGVTNQAFRDKEKPLAVRSANIVAARAVADAIRTSLGPRGMDKM
YEAST          -------------MSAKVPSNATFKNKEKPQEVRKANIIAARSVADAIRTSLGPKGMDKM
                             :.    : ::::::**  :* :** **::***********:*****

HUMAN          IQDGKGDVTITNDGATILKQMQVLHPAARMLVELSKAQDIEAGDGTTSVVIIAGSLLDSC
CtCCT4         IRSGKGETIITNDGSTMLKSMSVMHPTAKMLVQLSKAQDVEAGDGTTSVVVICGSLLGAA
YEAST          IKTSRGEIIISNDGHTILKQMAILHPVARMLVEVSAAQDSEAGDGTTSVVILTGALLGAA
               *: .:*:  *:*** *:**.* ::**.*:***::* *** **********:: *:**.:.

HUMAN          TKLLQKGIHPTIISESFQKALEKGIEILTDMSRPVELSDRETLLNSATTSLNSKVVSQYS
CtCCT4         DRLLQKGIHPSVISESFQRAAAAAVQVLHDMSQPIALTDTAALLQAANTSLSSKIVSQYS
YEAST          ERLLNKGIHPTIIADSFQSAAKRSVDILLEMCHKVSLSDREQLVRAASTSLSSKIVSQYS
                :**:*****::*::*** *   .:::* :*.: : *:*   *:.:*.***.**:*****

HUMAN          SLLSPMSVNAVMKVIDPATATSVDLRDIKIVKKLGGTIDDCELVEGLVLTQKVS--NSGI
CtCCT4         NLLGPMAVNAVTKTIDIKTADNVDLKNIRVIKKVGGTIEDSELVDGLVLTQPVIKSAGGP
YEAST          SFLAPLAVDSVLKISD-ENSKNVDLNDIRLVKKVGGTIDDTEMIDGVVLTQTAIKSAGGP
               .:*.*::*::* *  *  .: .***.:*:::**:****:* *:::*:**** .    .* 

HUMAN          TRVEKAKIGLIQFCLSAPKTDMDNQIVVSDYAQMDRVLREERAYILNLVKQIKKTGCNVL
CtCCT4         VRMEKARIGMIQFQLSPPKPDMENTIQVNDYRQMDKIVKEERQYLLNMVKKIKKAKCNVL
YEAST          TRKEKAKIGLIQFQISPPKPDTENNIIVNDYRQMDKILKEERAYLLNICKKIKKAKCNVL
               .* ***:**:*** :* ** * :* * *.** ***::::*** *:**: *:***: ****

HUMAN          LIQKSILRDALSDLALHFLNKMKIMVIKDIEREDIEFICKTIGTKPVAHIDQFTADMLGS
CtCCT4         FIQKSILRDAVNDLSLHFLQRLGILAVKDIERDEVEFICKSTGCKPIADIESFTEDKLGS
YEAST          LIQKSILRDAVNDLALHFLSKLNIMVVKDIEREEIEFLSKGLGCKPIADIELFTEDRLGS
               :*********:.**:****.:: *:.:*****:::**:.*  * **:*.*: ** * ***

HUMAN          AELAEEVNLNGSGKLLKITGCAS--PGKTVTIVVRGSNKLVIEEAERSIHDALCVIRCLV
CtCCT4         ADLVEEVHSAGSR-YVKVTGTKST--GKTVSVVVRGANSLILDEAERSLHDALCAVRCLV
YEAST          ADLVEEIDSDGSK-IVRVTGIRNNNARPTVSVVIRGANNMIIDETERSLHDALCVIRCLV
               *:*.**:.  **   :::**  .     **::*:**:*.::::*:***:*****.:****

HUMAN          KKRALIAGGGAPEIELALRLTEYSRTLSGMESYCVRAFADAMEVIPSTLAENAGLNPIST
CtCCT4         KKKALIAGGGAPEIEIAAQLNKQARALSGTEAICWKAFADAMEVIPTTLAENAGLNPIKV
YEAST          KERGLIAGGGAPEIEISRRLSKEARSMEGVQAFIWQEFASALEVIPTTLAENAGLNSIKV
               *::.***********:: :*.: :*::.* ::   : **.*:****:********* *..

HUMAN          VTELRNRHAQGEKTAGINVRKGGISN-ILEELVVQPLLVSVSALTLATETVRSILKIDDV
CtCCT4         VTDLRHRHEMGEKNAGVSIKSGGVSSDITKENVLQPLLVSTSAIELAAETVKMILRIDDI
YEAST          VTELRSKHENGELNDGISVRRSGTTN-TYEEHILQPVLVSTSAITLASECVKSILRIDDI
               **:** :*  ** . *:.:: .* :.   :* ::**:***.**: **:* *: **:***:

HUMAN          VNTR
CtCCT4         ALSR
YEAST          AFSR
               . :*
```

Alignment Scores

1: HUMAN	100.00	62.00	58.78
2: CtCCT4	62.00	100.00	65.78
3: YEAST	58.78	65.78	100.00

CCT5

```
YEAST         MAARPQQPPMEMPDLSNAIVAQDEMGRPFIIVKDQGNKKRQHGLEAKKSHILAARSVASI
CtCCT5        -------MGSMNIDLSNATVMKDEQGRPFIVVRDQGKKKRQHGNEAVRAHILAARTVANI
HUMAN         -------------MASMGTLAFDEYGRPFLIIKDQDRKSRLMGLEALKSHIMAAKAVANT
                             * . :  ** ****::::**..*.*  * ** ::**:**::**.

YEAST         IKTSLGPRGLDKILISPDGEITITNDGATILSQMELDNEIAKLLVQLSKSQDDEIGDGTT
CtCCT5        IKTSLGPRGLDKILISPDGDITVTNDGATILQQMEITNHVAKLLVELSKSQDDEIGDGTT
HUMAN         MRTSLGPNGLDKMMVDKDGDVTVTNDGATILSMMDVDHQIAKLMVELSKSQDDEIGDGTT
              ::*****.****:::. **::*:********. *:: :.:***:*:**************

YEAST         GVVVLASALLDQALELIQKGIHPIKIANGFDEAAKLAISKLEETCDDISASNDELFRDFL
CtCCT5        GVVVLAGALLEQAAELIDKGIHPIRIADGYDQACDIACAELDRISDVIEFDR--ENTENL
HUMAN         GVVVLAGALLEEAEQLLDRGIHPIRIADGYEQAARVAIEHLDKISDSVLVDI--KDTEPL
              ******.***::* :*:::*****:**:*:::*. :*  .*:. .* :  .      : *

YEAST         LRAAKTSLGSKIVSKDHDRFAEMAVEAVINVMDKDRKDVDFDLIKMQGRVGGSISDSKLI
CtCCT5        IKVARTSLGSKIVSKAHDQFAKIAVDAVLSVADLERKDVDFDLIKVDGKVGGSLEDTMLV
HUMAN         IQTAKTTLGSKVVNSCHRQMAEIAVNAVLTVADMERRDVDFELIKVEGKVGGRLEDTKLI
              ::.*:*:****:*.. * ::*::**:**:.* * :*:****:***::*:*** :.*: *:

YEAST         NGVILDKDFSHPQMPKCVLPKEGSDGVKLAILTCPFEPPKPKTKHKLDISSVEEYQKLQT
CtCCT5        KGVIIDKDFSHPQMPSEV------RDAKIAILTCAFEPPKPKTKHKLEISTVEEFKKLQN
HUMAN         KGVIVDKDFSHPQMPKKV------EDAKIAILTCPFEPPKPKTKHKLDVTSVEDYKALQK
              :***:**********. *       ..*:***** ************::::**::: **.

YEAST         YEQDKFKEMIDDVKKAGADVVICQWGFDDEANHLLLQNDLPAVRWVGGQELEHIAISTNG
CtCCT5        YEREKFVEMIQQIKDAGANLAICQWGFDDEANHLLLQNNLPAVRWVGGPEIELIAIATNG
HUMAN         YEKEKFEEMIQQIKETGANLAICQWGFDDEANHLLLQNNLPAVRWVGGPEIELIAIATGG
              **::** ***:::*.:**::.*****************:********* *:* ***:*.*

YEAST         RIVPRFQDLSKDKLGTCSRIYEQEFGTTKDRMLIIEQSKETKTVTCFVRGSNKMIVDEAE
CtCCT5        RIVPRFEDLRPEKLGTAGLVREITFGTTREKMLVIEECANTRAVTVFVRGSNKMIIDEAK
HUMAN         RIVPRFSELTAEKLGFAGLVQEISFGTTKDKMLVIEQCKNSRAVTIFIRGGNKMIIEEAK
              ******.:*  :*** .. : *  ****:::**:**:. ::::** *:**.****::**:

YEAST         RALHDSLCVVRNLVKDSRVVYGGGAAEVTMSLAVSEEADKQRGIDQYAFRGFAQALDTIP
CtCCT5        RSLHDALCVVRNLVRDNRVVYGGGSAEVACSLAVEDAAVKTPGLEQYAMRAFAEALDTIP
HUMAN         RSLHDALCVIRNLIRDNRVVYGGGAAEISCALAVSQEADKCPTLEQYAMRAFADALEVIP
              *:***:***:***::*.*******:**:: :***.: * *   ::***:*.**:**:.**

YEAST         MTLAENSGLDPIGTLSTLKSKQLKEK--ISNIGVDCLGYGSNDMKELFVVDPFIGKKQQI
CtCCT5        MTLAENSGLNPIATLAEIKSQQVKDPTARGRVGVDCMGTGKNNMKEAFVIDPLIGKKQQL
HUMAN         MALSENSGMNPIQTMTEVRARQVKEM--NPALGIDCLHKGTNDMKQQHVIETLIGKKQQI
              *:*:****::** *:: ::::*:*:      :*:**:  *.*:**: .*:: :******:

YEAST         LLATQLCRMILKIDNVIISGKDEY--
CtCCT5        MLATQLCRMVLKVNNVIVSGSGEEEF
HUMAN         SLATQMVRMILKIDDIRKPGESEE--
               ****: **:**::::   *..*


Alignment Scores
1: YEAST        100.00    67.64    60.26
2: CtCCT5        67.64   100.00    67.84
3: HUMAN         60.26    67.84   100.00
```

CCT6

```
YEAST        -MSLQLLNPKAESLRRDAALKVNVTSAEGLQSVLETNLGPKGTLKMLVDGAGNIKLTKDG
CtCCT6       MSAAQLLNPKAESRRRQEALRVNISAGEGLQDVLKSNLGPMGTIKMLVDGAGQIKLTKDG
HUMAN        MAAVKTLNPKAEVARAQAALAVNISAARGLQDVLRTNLGPKGTMKMLVSGAGDIKLTKDG
               : : ******  * : ** **:::..***.**.:**** **:****.***:*******

YEAST        KVLLTEMQIQSPTAVLIARAAAAQDEITGDGTTTVVCLVGELLRQAHRFIQEGVHPRIIT
CtCCT6       NVLLREMQIQNPTAVMIARAATAQDDICGDGTTSVVLLVGELLKQADRYIQEGLHPRIIT
HUMAN        NVLLHEMQIQHPTASLIAKVATAQDDITGDGTTSNVLIIGELLKQADLYISEGLHPRIIT
             :*** ***** *** :**:.*:***:* *****: * ::****:**. :*.**:******

YEAST        DGFEIARKESMKFLDEFKISKTNLSNDREFLLQVARSSLLTKVDADLTEVLTPIVTDAVL
CtCCT6       DGFEIAKNEALKFLDKFKLPKD---IDRELLLNVARTSLSTKLSSSLAQHLTPSIVDAVL
HUMAN        EGFEAAKEKALQFLEEVKVSRE---MDRETLIDVARTSLRTKVHAELADVLTEAVVDSIL
             :*** *::::::**::.*: :     *** *::***:** **: :.*:: **  :.*::*

YEAST        SVYDAQADNLDLHMVEIMQMQHLSPKDTTFIKGLVLDHGGRHPDMPTRVKNAYVLILNVS
CtCCT6       AIYQP-PAKPDLHMIEIMKMQHRTASDTQLIRGLALDHGARHPDMPKRVENAYILTLNVS
HUMAN        AIKKQ-DEPIDLFMIEIMEMKHKSETDTSLIRGLVLDHGARHPDMKKRVEDAYILTCNVS
             :: .      **.*:***:*:* : .** :*:**.****.***** .**::**:*  ***

YEAST        LEYEKTEVNSGFFYSSADQRDKLAASERKFVDAKLKKIIDLKNEVCGMDPDKGFVIINQK
CtCCT6       LEYEKSEINSGFFYSSAEQRDKLVESERKFVDLKLKKIVELKKQVCGNDPNKNFVVINQK
HUMAN        LEYEKTEVNSGFFYKSAEEREKLVKAERKFIEDRVKKIIELKRKVCGD-SDKGFVVINQK
             *****:*:******.**::*:**. :****:: ::***::**.:***   :*.**:****

YEAST        GIDPMSLDVFAKHNILALRRAKRRNMERLQLVTGGEAQNSVEDLSPQILGFSGLVYQETI
CtCCT6       GIDPLSLDVLAKNGILALRRAKRRNMERLQLVCGGVAQNSVDDLTPDVLGWAGLVYEQQL
HUMAN        GIDPFSLDALSKEGIVALRRAKRRNMERLTLACGGVALNSFDDLSPDCLGHAGLVYEYTL
             ****:***.::*..*:************* *. ** * **.:**:*: ** :****:  :

YEAST        GEEKFTYVTENTDPKSCTILIKGSTHYALAQTKDAVRDGLRAVANVLKDKNIIPGAGAFY
CtCCT6       GEEKYTFVEDVKDPKSVTILIKGPNQHTITQVTDAVRDGLRSVYNCIVDKAVVPGAGAFH
HUMAN        GEEKFTFIEKCNNPRSVTLLIKGPNKHTLTQIKDAVRDGLRAVKNAIDDGCVVPGAGAVE
             ****:*:: . .:*:* *:**** .:::::* .********:* * : *  ::*****.

YEAST        IALSRYLRSANMNKLGAKGKTKTGIEAFAEALLVIPKTLVKNSGFDPLDVLAMVEDELDD
CtCCT6       VACAAHLRSDEF-LKAVKGKAKFGVEAFADALLVIPKTLAANAGLDVQDAVALLQD----
HUMAN        VAMAEALIK--H-KPSVKGRAQLGVQAFADALLIIPKVLAQNSGFDLQETLVKIQAE---
             :* :  * .      ..**::: *::***:***:***.*. *:*:*  :.:. ::

YEAST        AQDSDETRYVGVDLNIGDSCDPTIEGIWDSYRVLRNAITGATGIASNLLLCDELLRAGRS
CtCCT6       --ELRAGNVAGIDLQTGQPMDPVLEGVFDSFRVLRNCIASSSSIASNLLLCDELLKARQM
HUMAN        --HSESGQLVGVDLNTGEPMVAAEVGVWDNYCVKKQLLHSCTVIATNILLVDEIMRAGMS
               .    . .*:**: *:    .  *::*.: * :: : ..: **:*:** **:::*

YEAST        TLKETPQ----------
CtCCT6       GRGGGPGGPGGMEGVEE
HUMAN        SLKG-------------
```

```
Alignment Scores
1: YEAST       100.00    63.93    57.55
2: CtCCT6       63.93   100.00    63.21
3: HUMAN        57.55    63.21   100.00
```

CCT7

```
HUMAN           ----MMPTPVILLKEGTDSSQGIPQLVSNISACQVIAEAVRTTLGPRGMDKLIVDGRGKA
CtCCT7          MAFAGQPPMIVVLKEGTDTSQGKGQILSNINACLAVQSTIKSTLGPYGGDLLLVDQNGKQ
YEAST           MNFGSQTPTIVVLKEGTDASQGKGQIISNINACVAVQEALKPTLGPLGSDILIVTSNQKT
                         :::******:***  *::***.** .: .::: **** * * *:*  . *

HUMAN           TISNDGATILKLLDVVHPAAKTLVDIAKSQDAEVGDGTTSVTLLAAEFLKQVKPYVEEGL
CtCCT7          TITNDGATVMKLLDIVHPAARILVDIARSQDAEVGDGTTSVVVLAGEILKEIKEHVEAGV
YEAST           TISNDGATILKLLDVVHPAAKTLVDISRAQDAEVGDGTTSVTILAGELMKEAKPFLEEGI
                **:*****::****:*****: ****:::************.:**.*::*: * .:* *:

HUMAN           HPQIIIRAFRTATQLAVNKIKEIAVTVKKADKVEQRKLLEKCAMTALSSKLISQQKAFFA
CtCCT7          STQIIIKGLRKAASMAVNKIREVAINA---EEGDRIDTLHKLAATAMTSKLIKRNSDFFT
YEAST           SSHLIMKGYRKAVSLAVEKINELAVDITS-EKSSGRELLERCARTAMSSKLIHNNADFFV
                  ::*::. *.*..:**:**.*:*:     :: .  . *.: * **::**** .:  **.

HUMAN           KMVVDAVMMLD-DLLQLKMIGIKKVQGGALEDSQLVAGVAFKKTFSYAGFEMQPKKYHNP
CtCCT7          KMVVEAVLSLDQDDLNEKLIGMKKIPGGSLTDSIFVKGVAFKKTFSYAGFEQQPKKFVKP
YEAST           KMCVDAVLSLDRNDLDDKLIGIKKIPGGAMEESLFINGVAFKKTFSYAGFEQQPKKFNNP
                ** *:**: ** : *: *:**:**: **:: :* :: ************** ****: :*

HUMAN           KIALLNVELELKAEKDNAEIRVHTVEDYQAIVDAEWNILYDKLEKIHHSGAKVVLSKLPI
CtCCT7          KICCLNVELELKAEKDNAEVRVEQVSEYQAIVDAEWQIIYNKLEAIYKTGAKVVLSKLPI
YEAST           KILSLNVELELKAEKDNAEVRVEHVEDYQAIVDAEWQLIFEKLRQVEETGANIVLSKLPI
                **  ***************:**. *.:*********:::::**. : .:**::*******

HUMAN           GDVATQYFADRDMFCAGRVPEEDLKRTMMACGGSIQTSVNALSADVLGRCQVFEETQIGG
CtCCT7          GDLATQYFADRDIFCAGRVTSEDMERVIQATGATIQSTCSDIRPEHLGTCGLFEERQIGG
YEAST           GDLATQFFADRNIFCAGRVSADDMNRVIQAVGGSIQSTTSDIKPEHLGTCALFEEMQIGS
                **:***:****::******  :*::*.: * *.:**:: . :  : ** * :*** ***.

HUMAN           ERYNFFTGCPKAKTCTFILRGGAEQFMEETERSLHDAIMIVRRAIKNDSVVAGGGAIEME
CtCCT7          ERFNFFEDCPEAKTCTLVLRGGAEQFIAEVERSLHDAIMIVKRAIKNKTTVAGGGATEME
YEAST           ERYNLFQGCPQAKTCTLLLRGGAEQVIAEVERSLHDAIMIVKRALQNKLIVAGGGATEME
                **:*:* .**:*****::*******.: *.***********:**::*.  ****** ***

HUMAN           LSKYLRDYSR-TIPGKQQLLIGAYAKALEIIPRQLCDNAGFDATNILNKLRARHAQGGTW
CtCCT7          VSAYLHRYADQTVRNKQQAIIKNFAKALEIIPRQLCDNAGFDATDILNRLRVEHRRGNIW
YEAST           VSKCLRDYSK-TIAGKQQMIINAFAKALEVIPRQLCENAGFDAIEILNKLRLAHSKGEKW
                :*  *: *:  *: .*** :*  :*****:******:****** :***:**  * :*  *

HUMAN           YGVDINNEDIADNFEAFVWEPAMVRINALTAASEAACLIVSVDETIKNPRSTVD-----A
CtCCT7          AGVDFQNEGVADMMEKFVWEPALVKINAINAATEAACLILSVDETIRNEESKTPPAPGSK
YEAST           YGVVFETENIGDNFAKFVWEPALVKINALNSATEATNLILSVDETITNKGSESANAGMMP
                 ** ::.*.:.* :  ******:*:***:.:*:**: **:****** *  *

HUMAN           PTA--AGRGRGRGRPH-
CtCCT7          PARGGAGRGRGRGMPRR
YEAST           P----QGAGRGRGMPM-
                *     * ***** *
```

```
Alignment Scores
1: HUMAN         100.00    63.15    63.15
2: CtCCT7         63.15   100.00    68.25
3: YEAST          63.15    68.25   100.00
```

CCT8

```
HUMAN       MALHVPKAPGFAQMLKEGAKHFSGLEEAVYRNIQACKELAQTTRTAYGPNGMNKMVINHL
CtCCT8      MSLSIPGAPN-AGLFKQGYNSYDSEDGAVLRNIDACRAISSTVQTSLGPYGRNKVVINHL
YEAST       MSLRLPQNPN-AGLFKQGYNSYSNADGQIIKSIAAIRELHQMCLTSMGPCGRNKIIVNHL
            *:* :*  *. * ::*:* : :.. :  : :.* * : : .   *: ** * **:::***

HUMAN       EKLFVTNDAATILRELEVQHPAAKMIVMASHMQEQEVGDGTNFVLVFAGALLELAEELLR
CtCCT8      GKMILTSDAATILRELDVVHPAAKLLVMASQQQEAEMGDATNLVIVLAGELLRKAEDLLR
YEAST       GKIIITNDAATMLRELDIVHPAVKVLVMATEQQKIDMGDGTNLVMILAGELLNVSEKLIS
             *:::*.****:****:: ***.*::***:. *: ::**.**:*:::** **. :*.*:

HUMAN       IGLSVSEVIEGYEIACRKAHEILPNLVCCSAKNLRDIDEVSSLLRTSIMSKQYGNEVFLA
CtCCT8      MGLKTSDIVNGYERAQKIALDALEELEVDKVEDLRNPEELKKALRTVIASKQNGSEDFLA
YEAST       MGLSAVEIIQGYNMARKFTLKELDEMVVGEITDKNDKNELLKMIKPVISSKKYGSEDILS
            :**.. ::::**: * : : . * ::   .  : .: :*: . ::  * **: *.* :*:

HUMAN       KLIAQACVSIFPDS------GHFNVDNIRVCKILGSGISSSSVLHGMVFKKETEGDVTS-
CtCCT8      GLVAEAVLSVLPKN-----PVNFNVDNVRVVKIMGGSLDQSRVVRGMVFNKEPDGAVKK-
YEAST       ELVSEAVSHVLPVAQQAGEIPYFNVDSIRVVKIMGGSLSNSTVIKGMVFNREPEGHVKSL
             *:::*   ::*          ****.:** **:*..:..* *::****::* :* *..

HUMAN       --VKDAKIAVYSCPFDGMITETKGTVLIKTAEELMNFSKGEENLMDAQVKAIADTGANVV
CtCCT8      --ARKAKVGVFTCPIDISQTETKGTVLLHNAKEMLNFSKGEEERLEAQIKELHDVGLRVV
YEAST       SEDKKHKVAVFTCPLDIANTETKGTVLLHNAQEMLDFSKGEEKQIDAMMKEIADMGVECI
               :. *:.*::**:*   ********::.*:*:::******: ::* :* : * * . :

HUMAN       VTGGKVADMALHYANKYNIMLVRLNSKWDLRRLCKTVGATALPRLTPPVLEEMGHCDSVY
CtCCT8      VAGSTVGELAMHYLNRYGILVIKIFSKFELRRLCRVVGATPLARLGAPMPDEMGTIDVVE
YEAST       VAGAGVGELALHYLNRYGILVLKVPSKFELRRLCRVCGATPLPRLGAPTPEELGLVETVK
            *:*. *.::*:** *:*.*::::: **::*****:. *** * **  *  :*:*  : *

HUMAN       LSEVGDTQVVVFKHEK-EDGAISTIVLRGSTDNLMDDIERAVDDGVNTFKVLTR--DKRL
CtCCT8      TQEIGGDRVTVFRQED-EATRTATIVLRGATQNHLDDLERAVDDGVNVIKAITK--DARL
YEAST       TMEIGGDRVTVFKQEQGEISRTSTIILRGATQNNLDDIERAIDDGVAAVKGLMKPSGGKL
              *:*. :*.**::*. *    :**:***:*:* :**:***:**** ..* : :  . :*

HUMAN       VPGGGATEIELAKQITSYGETCPGLEQYAIKKFAEAFEAIPRALAENSGVKANEVISKLY
CtCCT8      VPGAGATEIELVDRIQAAADKTPGLAQYSIKKYGEAFEVVPRTLAESAGLDATEVVSRLY
YEAST       LPGAGATEIELISRITKYGERTPGLLQLAIKQFAVAFEVVPRTLAETAGLDVNEVLPNLY
            :**.******* .:*   .:  *** * :**::. ***.:**:***.:*:...**: .**

HUMAN       AVHQEGN--------KNVGLDIEAE-VPAVKDMLEAGILDTYLGKYWAIKLATNAAVTVL
CtCCT8      AAHQK-------KDGWTTGVDIENQDNTGVLDAEDEGILDLLSSKQWAIKLATEAARTVL
YEAST       AAHNVTEPGAVKTDHLYKGVDIDGESDEGVKDIREENIYDMLATKKFAINVATEAATTVL
            *.*:              *:**: :   .* *  : .* *    * :**::**:** ***

HUMAN       RVDQIIMAKPAGGPKPPSGKK--DWDDDQND
CtCCT8      SVDQIIVARQAGGPKPP-GPN-PNWDED---
YEAST       SIDQIIMAKKAGGPRAPQGPRPGNWDQED--
             :****:*: ****: * * .  :**::
```

```
Alignment Scores
1: HUMAN       100.00    52.50    47.52
2: CtCCT8       52.50   100.00    60.15
3: YEAST        47.52    60.15   100.00
```
