# Supplementary material for: Asymmetry in the function and dynamics of the cytosolic group II chaperonin CCT/TRiC
Source: PLoS One. 2017 May 2;12(5):e0176054. doi: 10.1371/journal.pone.0176054 (PMC5413064; doi:10.1371/journal.pone.0176054)
Supplement: S3 Fig — (A) Alignment of the amino acid in ATP binding site across the eight subunits that comprise the CtCCT. The numbering refer to the ctCCT1 sequence. (B) CtCCT1 active site colored according to conservation scores calculated for CtCCT1 –CtCCT8 (PDF) [file pone.0176054.s003.pdf]

S3 Fig. ATP binding sites of CtCCT subunits

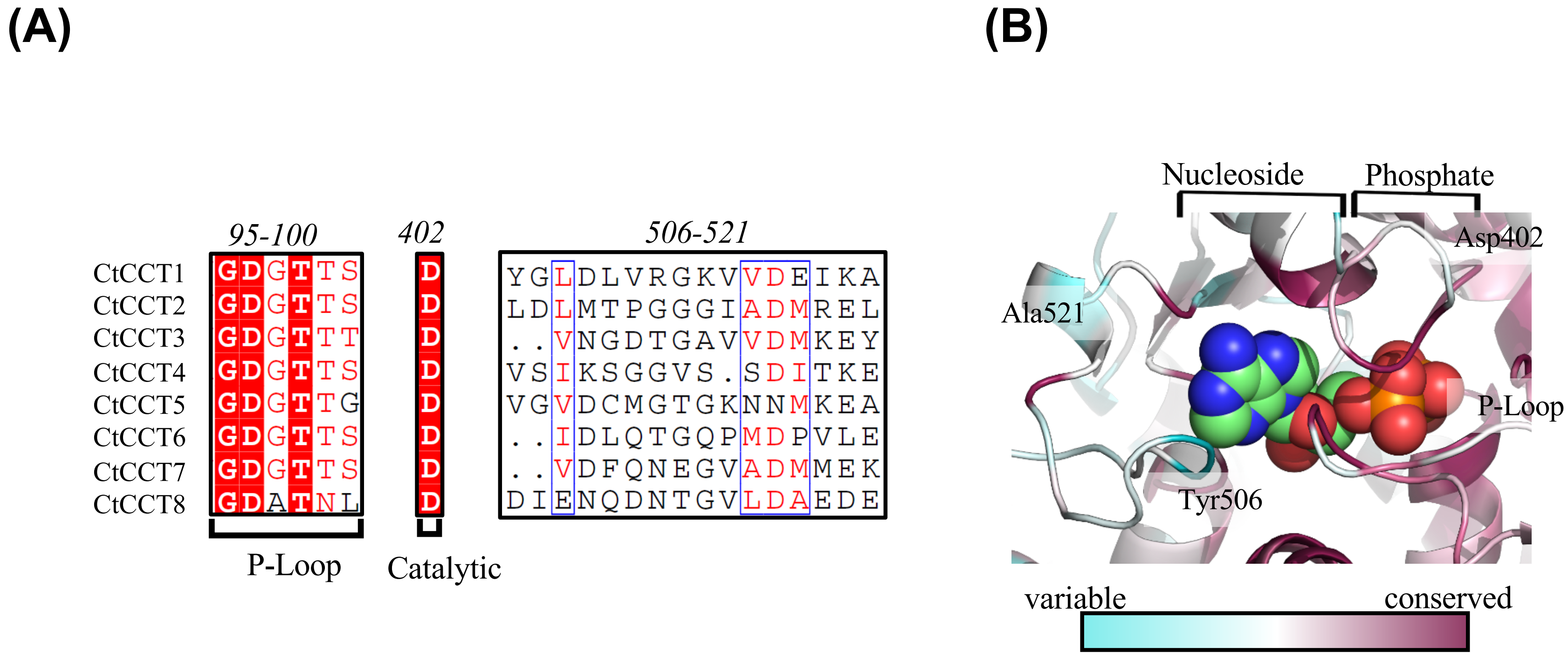

(A) **Alignment of the amino acid in ATP binding site across the eight subunits that comprise the CtCCT. The numbering refer to the ctCCT1 sequence**

(B) **CtCCT1 active site colored according to conservation scores calculated for CtCCT1 – CtCCT8**
