# Supplementary figures and images for: Asymmetry in the function and dynamics of the cytosolic group II chaperonin CCT/TRiC
Source: PLoS One. 2017 May 2;12(5):e0176054. doi: 10.1371/journal.pone.0176054 (PMC5413064; doi:10.1371/journal.pone.0176054)

**S4 Fig. Plasmids constructed for expressing CtCCT**

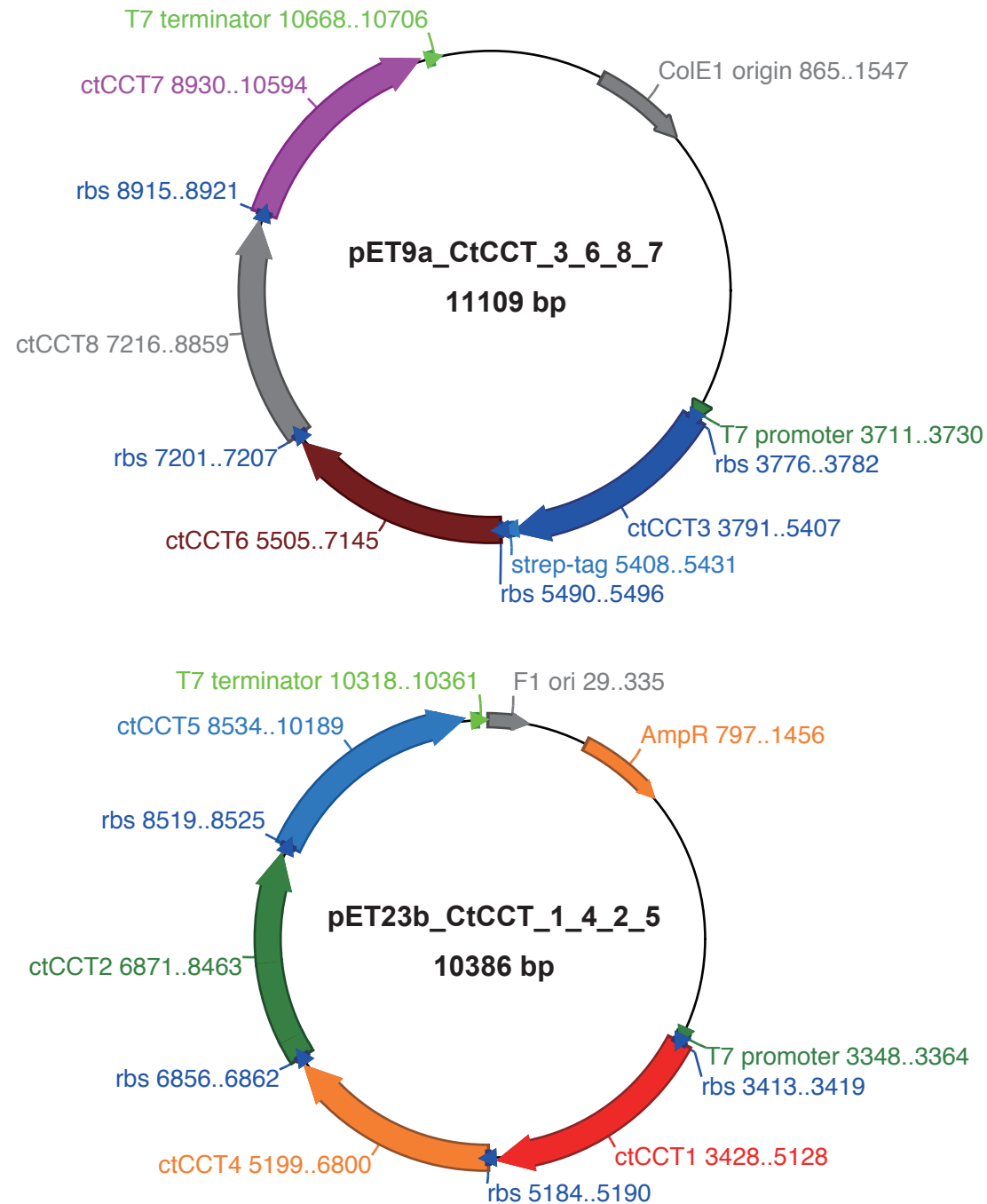

Supplement: S4 Fig — (PDF) [file pone.0176054.s004.pdf]

**S8 Fig.**

**Size exclusion chromatography of CtCCT variants with ATPase deficient mutant subunit**

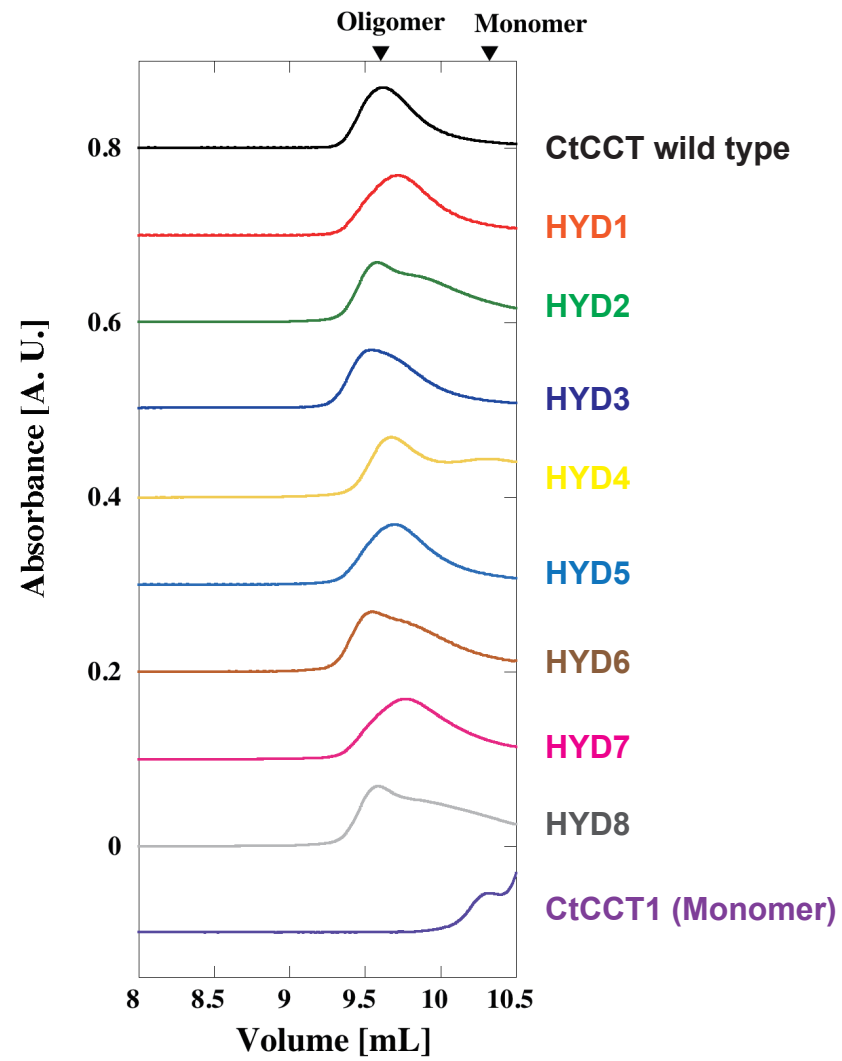

Supplement: S8 Fig — (PDF) [file pone.0176054.s008.pdf]
