# Supplementary material for: Asymmetry in the function and dynamics of the cytosolic group II chaperonin CCT/TRiC
Source: PLoS One. 2017 May 2;12(5):e0176054. doi: 10.1371/journal.pone.0176054 (PMC5413064; doi:10.1371/journal.pone.0176054)
Supplement: S5 Fig — (A) EM image of CtCCT homo-oligomer. Black bar represents 100 nm. (B) SEC-MALS analysis of CtCCT5. (PDF) [file pone.0176054.s005.pdf]

S5 Fig. Homo-oligomer formation of CtCCT5

(A)

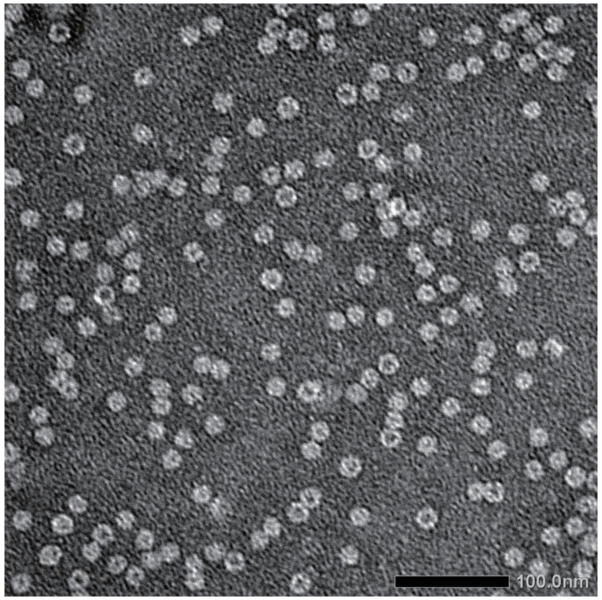

(B)

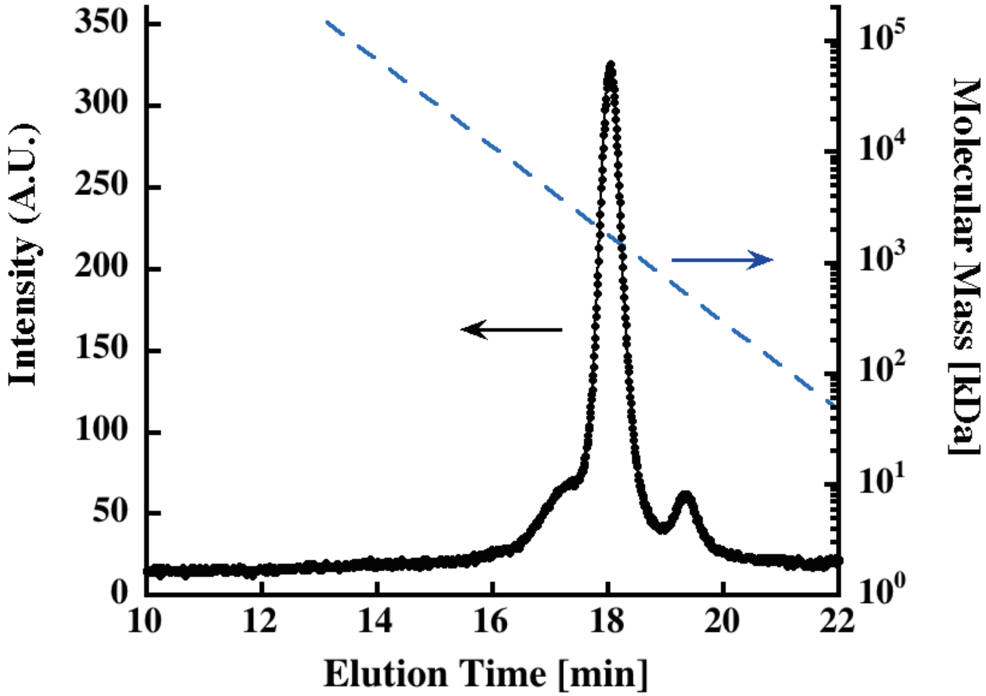

(A) EM image of CtCCT homo-oligomer. Black bar represents 100 nm.

(B) SEC-MALS analysis of CtCCT5.
