# Supplementary material for: Asymmetry in the function and dynamics of the cytosolic group II chaperonin CCT/TRiC
Source: PLoS One. 2017 May 2;12(5):e0176054. doi: 10.1371/journal.pone.0176054 (PMC5413064; doi:10.1371/journal.pone.0176054)
Supplement: S6 Fig — (PDF) [file pone.0176054.s006.pdf]

S6 Fig. Peptide coverage for CtCCT subunits detected by LC-MS/MS.

CtCCT1
Sequence Coverage: **63%**
Matched peptides shown in **Bold Red**

1 M**AAIFEQPRN GTLFLGGQKI SGADIRDQNV LATQAIANVV KSSFGPSGLD**

51 **KMMVDDIGDV TVTNDGATIL SLLDVEHPAG KILVDLAQQQ DKEVGDGTTS**

101 **VVLIAAELLK RANDLMK**NR**I HPTTIITGYR** LALREAVK**YM NEHVSIKVEN**

151 **LGR**ESLLNIA KTSMSSK**IIG ADADFFANMV VDAIQAVK**TT NNKNEVKYPV

201 K**AVNILK**AHG K**SATESMLIK** GYALNCTVAS QAMKTRITDA KIACLDMNLQ

251 KERMK**LGVQI TIDDPEQLEA IR**AR**EATMVL ER**IDMILK**AG ANVILTTK**GI

301 DDLCLKAFVE KGAMAVRRCK KEDLRRIAK**A TGATLLVTFS DLNGDEKFEP**

351 **SYLGHAEEVV QER**IADDECI LIKGTK**AHSS ASIILRGPNE FTLDEMER**SV

401 HDSLCAVKRT LESGSIVPGG GAVETALHIF LEEFAGTVGS R**EQLAIGEFA**

451 **QSLLVIPKTL AVNAAKDASE LVAQLR**SRHA LSQR**IQEGEG SEDEK**TIAKK

501 KAYK**NYGLDL VR**GK**VVDEIK AGVVEPSISK** IRQLKSATEA CIAIMR**IDTL**

551 **IKLDPEPQPE DDGHDH**

CtCCT2

Sequence Coverage: **53%**

Matched peptides shown in **Bold Red**

1 M**SSFSPTQIF EEGTTEEKGE NARLSAFVGA IAVGDLVK**ST LGPKGMDK**IL**

51 **QSASTGEIMV TNDGATILKS IALDNAAAKV LVNISKVQDD EVGDGTTSVA**

101 **VLAAELLR**EA EKLVNQK**IHP QTIIEGYRIA SQAALKALEA SAVDHSNNPE**

151 **QFKK**DLQAIA RTTLSSKVLA QDRDHFAK**LA VEAVLR**LKGS **SDLSHIQIIK**

201 KAGGKLCESY LDEGFILDKK IGVNQPKRLE KAK**ILIANTP MDTDKVK**IFG

251 ARLKVSSTSK **LAELER**AERE KMKAKVEKIK AHGINCFINR QLIYNWPEQL

301 FTDAGIMSIE HADFDGIER**L ALVTGGEIAS TFDHPEQVK**L GYCDLIEEIM

351 IGEDTLIKFS GVAAGEACTI VLR**GATDQLL DEAERSLHDA LAVLSQMVK**E

401 PRTILGGGCA EMIMAKAVEA AATRVEGKK**Q TAVGAFAVAL RQLPTILADN**

451 **AGLDSGDLVA R**LRKAIYDGL TTYGLDLMTP GGGIADMR**EL GVIESYK**LKR

501 **AVVSSASEAA ELLLRVDDII R**AAPRKREKH

CtCCT3

Sequence Coverage: **69%**

Matched peptides shown in **Bold Red**

1 **MQAPVLVMNT QSGDR**TTGR**R AQISNIAAAK TVADIIR**SCL GPKAMLK**MLL**

51 **DPMGGIVLTN DGHAILREIE VSHPAAKSMI ELSRTQDEEV GDGTTTVIIL**

101 **AGEILAQALP QLERNIHPVN IIAAFK**RALK **DALEIIEEIS MPIDVNDDK**A

151 MYK**LINASIG TK**FVSRWSDL MCSLALKAVR **TVTWEANGKR EVDIK**RYAR**V**

201 **EKVPGGEIED SRVLDGVMLN K**DITHPKMRR RIENPRIVLL DCPLEYK**KGE**

251 **SQTNIEITK**E DDWNR**ILQIE EEQVKAMCDA ILAVKPDLVI TEKGVSDLAQ**

301 **HYLMKANVTA LR**RVRKTDNN RIAR**AVGATI VNR**VEDLQES DVGTGCGLFE

351 IEKIGDEYFT FLTQCKNPKA CTILLR**GPSK DVLNEIERNL QDAMGVARNV**

401 **MFHPRLSPGG GATEMAVSVR LAQMARQIEG VQQWPYKAVA EALEVIPRTL**

451 **VQNAGK**SPVR **VLTELR**AKHA EGK**HSFGVNG DTGAVVDMKE YGVWEPQAIK**

501 VQSMKTAIEA ACLLLRVDDI CSAKK**AQPGV GTGGAAQDDW SHPQFEK**

CtCCT4

Sequence Coverage: **46%**

Matched peptides shown in **Bold Red**

1 M**ATATQAGSG VTNQAFR**DKE KPLAVRSANI VAAR**AVADAI RTSLGPR**GMD

51 KMIRSGK**GET IITNDGSTML K**SMSVMHPTA K**MLVQLSK**AQ DVEAGDGTTS

101 VVVICGSLLG AADRLLQK**GI HPSVISESFQ R**AAAAAVQVL HDMSQPIALT

151 DTAALLQAAN TSLSSKIVSQ YSNLLGPMAV NAVTKTIDIK **TADNVDLK**NI

201 RVIKK**VGGTI EDSELVDGLV LTQPVIK**SAG GPVRMEKARI GMIQFQLSPP

251 KPDMENTIQV NDYRQMDKIV KEERQYLLNM VKKIKKAKCN VLFIQKSILR

301 **DAVNDLSLHF LQRLGILAVK** DIERDEVEFI CKSTGCKPIA DIESFTEDK**L**

351 **GSADLVEEVH SAGSR**YVKVT GTKSTGK**TVS VVVRGANSLI LDEAERSLHD**

401 **ALCAVRCLVK** KK**ALIAGGGA PEIEIAAQLN K**QARALSGTE AICWK**AFADA**

451 **MEVIPTTLAE NAGLNPIKVV TDLR**HRHEMG EKNAGVSIK**S GGVSSDITKE**

501 **NVLQPLLVST SAIELAAETV K**MILR**IDDIA LSR**

CtCCT5

Sequence Coverage: **46%**

Matched peptides shown in **Bold Red**

1 M**GSMNIDLSN ATVMK**DEQGR PFIVVRDQGK KKRQHGNEAV R**AHILAAR**TV

51 ANIIK**TSLGP** RGLDK**ILISP DGDITVTNDG ATILQQMEIT NHVAK**LLVEL

101 SK**SQDDEIGD GTTGVVVLAG ALLEQAAELI DKGIHPIR**IA DGYDQACDIA

151 CAELDR**ISDV IEFDRENTEN LIK**VARTSLG SKIVSKAHDQ FAK**IAVDAVL**

201 **SVADLERKDV DFDLIK**VDGK **VGGSLEDTML VK**GVIIDK**DF SHPQMPSEVR**

251 DAKIAILTCA FEPPKPKTK**H KLEISTVEEF KKLQNYER**EK **FVEMIQQIK**D

301 AGANLAICQW GFDDEANHLL LQNNLPAVR**W VGGPEIELIA IATNGR**IVPR

351 FEDLRPEK**LG TAGLVREITF GTTR**EKMLVI EECANTR**AVT VFVR**GSNK**MI**

401 **IDEAK**RSLHD ALCVVRNLVR DNRVVYGGGS AEVACSLAVE DAAVKTPGLE

451 QYAMRAFAEA LDTIPMTLAE NSGLNPIATL AEIKSQQVKD PTARGRVGVD

501 CMGTGKNNMK **EAFVIDPLIG KK**QQLMLATQ LCRMVLK**VNN VIVSGSGEEE**

551 **F**

CtCCT6
Sequence Coverage: **58%**
Matched peptides shown in **Bold Red**

1 M**SAAQLLNPK** AESRRRQEAL R**VNISAGEGL** **QDVLKSNLGP** **MGTIKMLVDG**

51 **AGQIK**LTK**DG** **NVLLREMQIQ** **NPTAVMIARA** **ATAQDDICGD** **GTTSVVLLVG**

101 **ELLK**QADR**YI** **QEGLHPRIIT** **DGFEIAK**NEA LKFLDK**FKLP** **K**DIDR**ELLLN**

151 **VAR**TSLSTKL SSSLAQHLTP SIVDAVLAIY QPPAKPDLHM IEIMKMQHR**T**

201 **ASDTQLIRGL** **ALDHGARHPD** **MPK**RVENAYI LTLNVSLEYE K**SEINSGFFY**

251 **SSAEQRDKLV** **ESER**K**FVDLK** LK**KIVELK**KQ VCGNDPNK**NF** **VVINQKGIDP**

301 **LSLDVLAKNG** **ILALR**RAKRR NMERLQLVCG GVAQNSVDDL TPDVLGWAGL

351 VYEQQLGEEK **YTFVEDVKDP** **KSVTILIKGP** **NQHTITQVTD** **AVR**DGLRSVY

401 NCIVDKAVVP GAGAFHVACA AHLRSDEFLK AVKGKAK**FGV** **EAFADALLVI**

451 **PKTLAANAGL** **DVQDAVALLQ** **DELRAGNVAG** **IDLQTGQPMD** **PVLEGVFDSF**

501 **R**VLRNCIASS SSIASNLLLC DELLKARQMG R**GGGPGGPGG** **MEGVEE**

CtCCT7

Sequence Coverage: **61%**

Matched peptides shown in **Bold Red**

1 MAFAGQPPMI VVLKEGTDTS QGKGQILSNI NACLAVQSTI K**STLGPYGGD**

51 **LLLVDQNGKQ TITNDGATVM KLLDIVHPAA R**ILVDIAR**SQ DAEVGDGTTS**

101 **VVVLAGEILK** EIK**EHVEAGV STQIIIK**GLR K**AASMAVNK**I R**EVAINAEEG**

151 **DRIDTLHKLA ATAMTSK**LIK **RNSDFFTKMV VEAVLSLDQD DLNEK**LIGMK

201 **KIPGGSLTDS IFVK**GVAFK**K TFSYAGFEQQ PK**KFVKPKIC CLNVELELKA

251 EKDNAEVR**VE QVSEYQAIVD AEWQIIYNK**L EAIYKTGAKV VLSK**LPIGDL**

301 **ATQYFADR**DI FCAGR**VTSED MER**VIQATGA TIQSTCSDIR PEHLGTCGLF

351 EERQIGGERF NFFEDCPEAK TCTLVLR**GGA EQFIAEVERS LHDAIMIVK**R

401 AIKNK**TTVAG GGATEMEVSA YLHRYADQTV R**NKQQAIIKN FAK**ALEIIPR**

451 **QLCDNAGFDA TDILNR**LRVE HRR**GNIWAGV DFQNEGVADM MEKFVWEPAL**

501 **VKINAINAAT EAACLILSVD ETIR**NEESK**T PPAPGSKPAR** GGAGRGRGRG

551 MPRR

CtCCT8

Sequence Coverage: **80%**

Matched peptides shown in **Bold Red**

1 M**SLSIPGAPN AGLFKQGYNS YDSEDGAVLR** NIDACR**AISS TVQTSLGPYG**

51 **R**NK**VVINHLG KMILTSDAAT ILRELDVVHP AAKLLVMASQ QQEAEMGDAT**

101 **NLVIVLAGEL LRKAEDLLR**M GLK**TSDIVNG YER**AQK**IALD ALEELEVDKV**

151 **EDLRNPEELK** KALRTVIASK **QNGSEDFLAG LVAEAVLSVL PKNPVNFNVD**

201 **NVR**VVK**IMGG SLDQSR**VVR**G MVFNKEPDGA VK**KARKAK**VG VFTCPIDISQ**

251 **TETKGTVLLH NAKEMLNFSK** GEEER**LEAQI KELHDVGLRV VVAGSTVGEL**

301 **AMHYLNR**YGI LVIKIFSKFE LRRLCRVVGA TPLAR**LGAPM PDEMGTIDVV**

351 **ETQEIGGDRV TVFRQEDEAT RTATIVLRGA TQNHLDDLER AVDDGVNVIK**

401 AITKDAR**LVP GAGATEIELV DR**IQAAADKT PGLAQYSIK**K YGEAFEVVPR**

451 **TLAESAGLDA TEVVSR**LYAA HQK**KDGWTTG VDIENQDNTG VLDAEDEGIL**

501 **DLLSSK**QWAI K**LATEAARTV LSVDQIIVAR QAGGPKPPGP NPNWDED**
