# Supplementary material for: Asymmetry in the function and dynamics of the cytosolic group II chaperonin CCT/TRiC
Source: PLoS One. 2017 May 2;12(5):e0176054. doi: 10.1371/journal.pone.0176054 (PMC5413064; doi:10.1371/journal.pone.0176054)
Supplement: S7 Fig — (PDF) [file pone.0176054.s007.pdf]

S7 Fig. TEM images of CtCCT variants used for DXT experiment

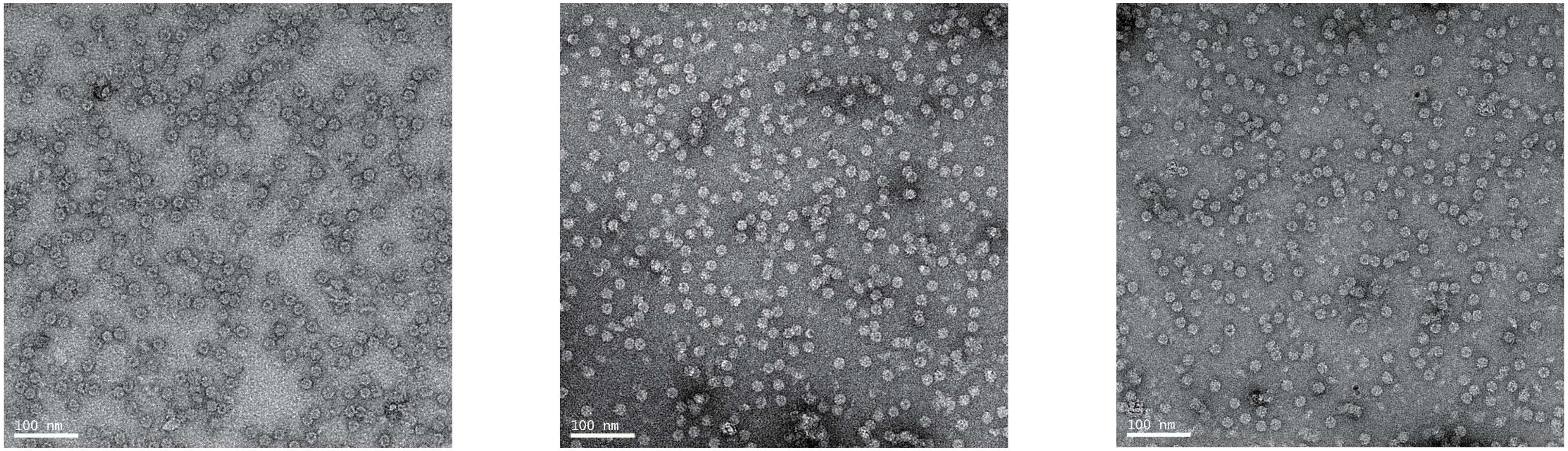

CtCCT^{C1C2} CtCCT^{C4C8} CtCCT^{C6C7}

The white bar represnts 100nm.
