# Supplementary material for: Asymmetry in the function and dynamics of the cytosolic group II chaperonin CCT/TRiC
Source: PLoS One. 2017 May 2;12(5):e0176054. doi: 10.1371/journal.pone.0176054 (PMC5413064; doi:10.1371/journal.pone.0176054)
Supplement: S9 Fig — (PDF) [file pone.0176054.s009.pdf]

S9 Fig.

TEM images of CtCCT variants with ATPase deficient mutant subunit

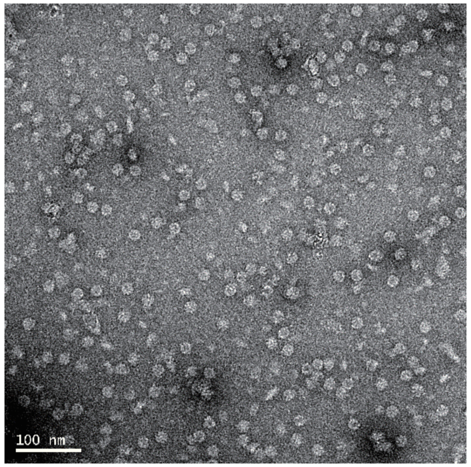

HYD1

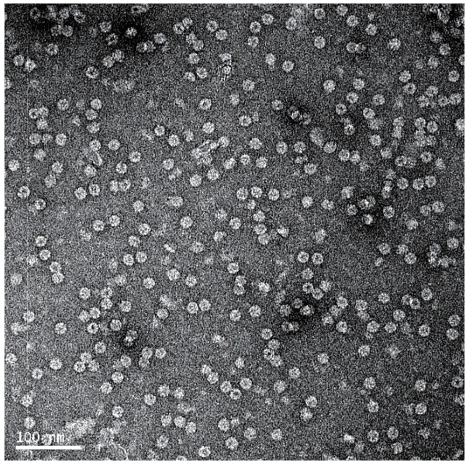

HYD2

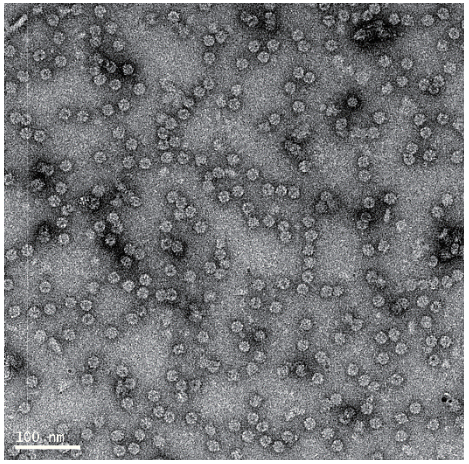

HYD3

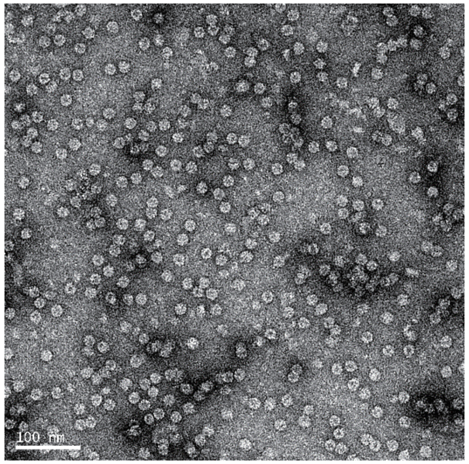

HYD4

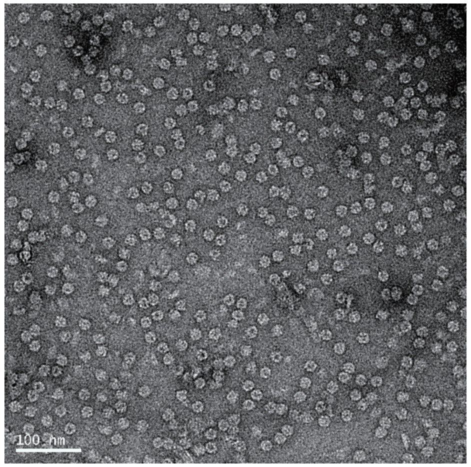

HYD5

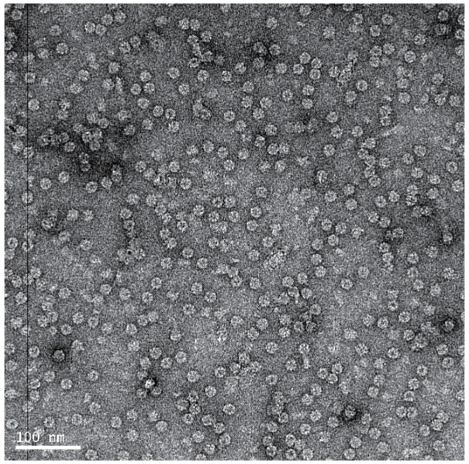

HYD6

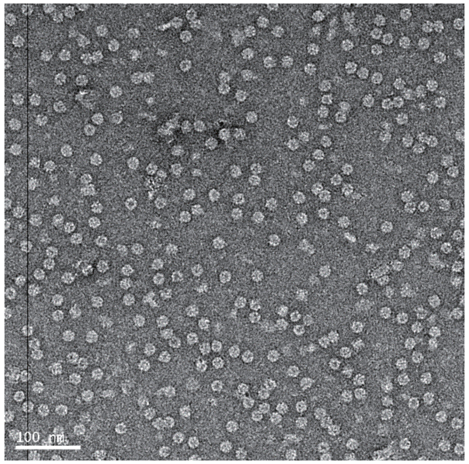

HYD7

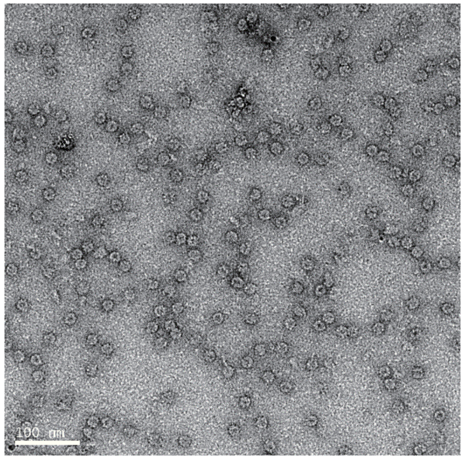

HYD8
