# Supplementary material for: Asymmetry in the function and dynamics of the cytosolic group II chaperonin CCT/TRiC
Source: PLoS One. 2017 May 2;12(5):e0176054. doi: 10.1371/journal.pone.0176054 (PMC5413064; doi:10.1371/journal.pone.0176054)
Supplement: S1 Table — (PDF) [file pone.0176054.s010.pdf]

S1 Table. Primers used for cloning CtCCT subunits

Subunit		Sequence		Enzyme
CtCCT1	5'	ggatatccatATGGCCGCCATTTTTGAG	3'	NdeI
	5'	ggaattcCTAATGATCGTGTCCATCATCTTCC	3'	EcoRI
CtCCT2	5'	ggatatccatATGTCGTCGTTCAGCCCC	3'	NdeI
	5'	ggaattcTTAGTGCTTCTCCCGCTTCC	3'	EcoRI
CtCCT3	5'	ggatatccatATGCAGGCCCCTGTCTTG	3'	NdeI
	5'	ggaattcCTAATCATCCTGCGCAGCAC	3'	EcoRI
CtCCT4	5'	ggatatccatATGGCGACCGCAACACAA	3'	NdeI
	5'	ggaattcTTACCTGCTCAGGGCGATATCG	3'	EcoRI
CtCCT5	5'	ggatatccatATGGGTTCCATGAATATCGACCTGTC	3'	NdeI
	5'	ggaattcTTAAAATTCCTCCTCACCAGATCCGG	3'	EcoRI
CtCCT6	5'	ggatatccatATGTCAGCAGCACAGCTCCT	3'	NdeI
	5'	cccaagcttTTACTCCTCAACACCCTCCATTC	3'	HindIII
CtCCT7	5'	ggatatccatATGGCGTTCGCGGGGCAAC	3'	NdeI
	5'	ggaattcTCACCGCCGCGGCATG	3'	EcoRI
CtCCT8	5'	ggatatccatATGTCCCTTAGCATTCCCGGCG	3'	NdeI
	5'	ggaattcTCAGTCTTCGTCCCAGTTCGGGTT	3'	EcoRI
