# Supplementary material for: Asymmetry in the function and dynamics of the cytosolic group II chaperonin CCT/TRiC
Source: PLoS One. 2017 May 2;12(5):e0176054. doi: 10.1371/journal.pone.0176054 (PMC5413064; doi:10.1371/journal.pone.0176054)
Supplement: S2 Table — (PDF) [file pone.0176054.s011.pdf]

S2 Table. The median values and the pairwise p-values for the single molecule distributions in Fig. 4A calculated by the Wilcoxon rank-sum test

CtCCT^{C1C2}

	Median (10^{-3} rad/sec)	0-1 s	1-2 s	2-3 s	3-4 s
0-1 s	2.93		< .0001	< .0001	< .0001
1-2 s	2.58	< .0001		0.805	0.001
2-3 s	2.58	< .0001	0.805		0.001
3-4 s	2.40	< .0001	0.001	0.001	

CtCCT^{C6C7}

	Median (10^{-3} rad/sec)	0-1 s	1-2 s	2-3 s	3-4 s
0-1 s	2.81		0.931	0.005	0.048
1-2 s	2.86	0.931		0.006	0.056
2-3 s	2.59	0.005	0.006		0.372
3-4 s	2.81	0.048	0.056	0.372	
