# Supplementary material for: Asymmetry in the function and dynamics of the cytosolic group II chaperonin CCT/TRiC
Source: PLoS One. 2017 May 2;12(5):e0176054. doi: 10.1371/journal.pone.0176054 (PMC5413064; doi:10.1371/journal.pone.0176054)
Supplement: S3 Table — (PDF) [file pone.0176054.s012.pdf]

S3 Table. The pairwise p-values for the single molecule distributions in Fig. 4A calculated by the Wilcoxon rank-sum test

CtCCT^{C1C2}

	0-2 s	2-4 s	4-6 s
0-2 s		0.072	0.280
2-4 s	0.072		0.515
4-6 s	0.280	0.515	

CtCCT^{C4C8}

	0-2 s	2-4 s	4-6 s
0-2 s		< .0001	0.527
2-4 s	< .0001		< .0001
4-6 s	0.527	< .0001	

CtCCT^{C6C7}

	0-2 s	2-4 s	4-6 s
0-2 s		< .0001	< .0001
2-4 s	< .0001		0.027
4-6 s	< .0001	0.027	
